# Comparison of traditional and DNA metabarcoding samples for monitoring tropical soil arthropods (Formicidae, Collembola and Isoptera)

**DOI:** 10.1038/s41598-022-14915-2

**Published:** 2022-06-24

**Authors:** Yves Basset, Mehrdad Hajibabaei, Michael T. G. Wright, Anakena M. Castillo, David A. Donoso, Simon T. Segar, Daniel Souto-Vilarós, Dina Y. Soliman, Tomas Roslin, M. Alex Smith, Greg P. A. Lamarre, Luis F. De León, Thibaud Decaëns, José G. Palacios-Vargas, Gabriela Castaño-Meneses, Rudolf H. Scheffrahn, Marleny Rivera, Filonila Perez, Ricardo Bobadilla, Yacksecari Lopez, José Alejandro Ramirez Silva, Maira Montejo Cruz, Angela Arango Galván, Héctor Barrios

**Affiliations:** 1 ForestGEO, Smithsonian Tropical Research Institute, Apartado 0843-03092, Balboa, Ancon, Panamá, Panama; 2 Faculty of Science, University of South Bohemia, 370 05 Ceske Budejovice, Czech Republic; 3 Biology Centre of the Czech Academy of Sciences, Institute of Entomology, 370 05 Ceske Budejovice, Czech Republic; 4 Maestría de Entomología, Universidad de Panamá, 080814 Panama City, Republic of Panama; 5 Centre for Biodiversity Genomics, Biodiversity Institute of Ontario and Department of Integrative Biology, University of Guelph, 50 Stone Road East, Guelph, ON N1G2W1 Canada; 6 Centro de Biodiversidad y Descubrimiento de Drogas, Instituto de Investigaciones Científicas y Servicios de Alta Tecnología (INDICASAT-AIP), P.O. Box 0843-01103, Panamá 5, Panama; 7 Department of Biotechnology, Acharya Nagarjuna University, Guntur, Andhra Pradesh 522 510 India; 8 Departamento de Biología, Escuela Politécnica Nacional, Quito, Ecuador; 9 Centro de Investigación de la Biodiversidad y Cambio Climático, Universidad Tecnológica Indoamérica, EC170103 Quito, Ecuador; 10 Agriculture and Environment Department, Harper Adams University, Newport, TF10 8NB Shropshire UK; 11 Department of Ecology, Faculty of Science, Charles University, Vinicna 7, 128 44 Prague, Czech Republic; 12 Department of Ecology, Swedish University of Agricultural Sciences, P.O. Box 7044, 750 07 Uppsala, Sweden; 13 Department of Integrative Biology, University of Guelph, Guelph, ON N1G2W1 Canada; 14 Department of Biology, University of Massachusetts Boston, 100 Morrissey Blvd., Boston, MA 02125 USA; 15 CEFE, University of Montpellier, CNRS, EPHE, IRD, University Paul Valéry, Montpellier 3, Montpellier, France; 16 Laboratorio de Ecología y Sistemática de Microartrópodos, Departamento de Ecología y Recursos Naturales, Facultad de Ciencias, Universidad Nacional Autónoma de México, 04510 Mexico City, Mexico; 17 Unidad Multidisciplinaria de Docencia e Investigación, Facultad de Ciencias, Universidad Nacional Autónoma de México, Campus Juriquilla, Juriquilla 76230, Querétaro, Mexico; 18 Fort Lauderdale Research & Education Center, 3205 College Avenue, Davie, FL 33314 USA

**Keywords:** Entomology, Biodiversity, Community ecology, Molecular ecology, Population dynamics

## Abstract

The soil fauna of the tropics remains one of the least known components of the biosphere. Long-term monitoring of this fauna is hampered by the lack of taxonomic expertise and funding. These obstacles may potentially be lifted with DNA metabarcoding. To validate this approach, we studied the ants, springtails and termites of 100 paired soil samples from Barro Colorado Island, Panama. The fauna was extracted with Berlese-Tullgren funnels and then either sorted with traditional taxonomy and known, individual DNA barcodes (“traditional samples”) or processed with metabarcoding (“metabarcoding samples”). We detected 49 ant, 37 springtail and 34 termite species with 3.46 million reads of the COI gene, at a mean sequence length of 233 bp. Traditional identification yielded 80, 111 and 15 species of ants, springtails and termites, respectively; 98%, 37% and 100% of these species had a Barcode Index Number (BIN) allowing for direct comparison with metabarcoding. Ants were best surveyed through traditional methods, termites were better detected by metabarcoding, and springtails were equally well detected by both techniques. Species richness was underestimated, and faunal composition was different in metabarcoding samples, mostly because 37% of ant species were not detected. The prevalence of species in metabarcoding samples increased with their abundance in traditional samples, and seasonal shifts in species prevalence and faunal composition were similar between traditional and metabarcoding samples. Probable false positive and negative species records were reasonably low (13–18% of common species). We conclude that metabarcoding of samples extracted with Berlese-Tullgren funnels appear suitable for the long-term monitoring of termites and springtails in tropical rainforests. For ants, metabarcoding schemes should be complemented by additional samples of alates from Malaise or light traps.

## Introduction

Soil invertebrates provide many ecosystem services, such as carbon transformation and sequestration, soil formation and recycling of nutrients^1^. Yet, the fauna of the soil remains one of the least known components of the biosphere, and in the tropics, it is often considered a biotic frontier^2,3^. Despite an increase in research on the soil fauna during recent years, one key obstacle that remains is the taxonomic impediment.

What renders the monitoring of the tropical soil fauna more topical than ever is climate change. Current projections estimate a global increase in average temperatures of 0.2 °C per decade within the coming century^4^. Such change is impacting forests worldwide and threatening biodiversity and concomitant ecosystem services^5^. In tropical rainforests the effects of climate change on the soil fauna are basically unknown but are most likely significant^6^. For example, increasing tree mortality may greatly raise temperatures on the forest floor^7^. We may foresee substantial changes in the species composition and functioning of tropical soils^6^ but lack both the baselines and the tools for detecting impending changes.

In the context of global change, molecular methods offer promising tools for lifting some of the taxonomic impediments and thus allowing for sound and low-cost biological monitoring^8–11^. Molecular approaches present several advantages: first, individuals and species can often be identified by sequencing standard gene regions (DNA barcodes)—for animals most often their cytochrome *c* oxidase subunit I (COI) gene^12,13^. COI-based clusters allow for interim taxonomic nomenclature, by e.g., using the Barcode Index Number (BIN^14^). Second, samples including many individuals and species can be treated by bulk methods, and COI amplicons can be analyzed using high-throughput sequencing, “DNA metabarcoding” (hereafter “metabarcoding”^15,16^). This represents a powerful approach for screening numerous species-rich environmental samples and offers new solutions for spatial and temporal monitoring^9,16,17^, particularly for community studies of soil assemblages^10,18–22^. Yet, there is a major challenge: to date, only presence-absence data (prevalence in samples, as opposed to abundance) can be reliably retrieved with metabarcoding^17,23–27^.

Among arthropods, three taxa represent major components of the soil fauna in tropical rainforests: Formicidae (ants), Collembola (springtails) and Isoptera (termites). Ants represent an important proportion of animal biomass in tropical rainforests where, in addition to exerting a formidable predation pressure, they play critical roles in the maintenance and regeneration of the forest, such as soil turnover, nutrient cycling, plant protection and seed dispersal^28^. Springtails are detritivorous or fungivorous and play important roles in litter decomposition, the formation of soil microstructure, and the release of humic acids, which are crucial for plant development. With their considerable biomass, they also represent a crucial part in food webs^29^. Termites are another group of dominant and abundant detritivorous arthropods in the tropics, which play a capital role as an ecosystem engineer by participating in the formation of the soil structure and the creation and maintenance of habitats for other soil organisms^1,30^.

The ForestGEO Arthropod Initiative represents a unique effort to monitor arthropods in tropical rainforests. Arthropod surveys are performed within permanent forest plots monitored by the Forest Global Earth Observatories (ForestGEO; http://www.forestgeo.si.edu/^31^). In Panama, the ForestGEO Arthropod Initiative has been monitoring several focal taxa on Barro Colorado Island (BCI) since 2009^32^. Decades of research on BCI and recent syntheses indicate that more than 200 ant, 100 springtail and 60 termite species may occur in the soil and litter on the island^33^. However, the long-term monitoring of at least the common species of these taxa remains challenging.

For social insects, such as ants or termites, colony census may represent the best estimate of population levels^34^. However, accurate censuses of often small and concealed colonies are problematic in tropical rainforests. In this case, surveying individual workers is more feasible. Nonetheless, since ants and termites have strongly aggregated distributions, abundance data and estimates of variability may be highly dependent on the distance to colony and related factors, such as behavior and speed. Hence, species prevalence in samples (presence-absence data) is often used as a surrogate of species abundance^34,35^. Here, we propose to use species prevalence to estimate annual population indices based on metabarcoding data^36^.

To provide an efficient, reliable, unbiased and sensitive monitoring protocol, the molecular methods applied should essentially fulfill a series of criteria^9,10,17,36^. First, metabarcoding techniques should be able to resolve the species composition of samples of known taxonomic content. Second, DNA-based data on species prevalence (i.e., presence-absence data) should ideally reflect some aspect of species abundance in the samples^37^. Third, metabarcoding techniques should resolve ecological patterns in community composition, such as seasonal (or annual) shifts in species prevalence. Fourth, any association between species biomass and DNA read frequencies^38^ would add to the information value of metabarcoding samples. Fifth, the prevalence data gained from metabarcoding should neither be biased towards “false positive” records (i.e., species detected in the sample although they are actually absent) or “false negative” records (i.e., species not detected although they are present in the sample^39–42^). Sixth, the data delivered by metabarcoding should be equally reliable across taxa, i.e., free of taxonomic biases.

To validate the suitability of metabarcoding for next-generation monitoring of soil arthropods in the tropics^43^, we compared the fauna of paired soil samples either sorted morphologically with traditional taxonomy and known barcodes (“traditional samples”) or processed with metabarcoding (“metabarcoding samples”). We focused on ants, springtails and termites because (1) they are dominant arthropod groups in tropical soils, and (2) we previously generated local barcode libraries for these groups. Our emphasis was also on common as opposed to rare species, as only the former can realistically be well monitored in tropical rainforests^44^. As a basis for the reliable biomonitoring of the tropical soil fauna, we ask:

1. Are the species richness and faunal composition of focal groups (ants, springtails and termites) of traditional samples similar to that of metabarcoding samples, at least when considering common species?
2. In this local study, can species prevalence in metabarcoding samples (i.e., the number of samples in which a species is present) be used as a proxy for species abundance in traditional samples, at least for common species?
3. Do seasonal shifts in species prevalence and composition between the dry and wet season detected with traditional samples match similar seasonal shifts for metabarcoding samples?
4. Is there any association between species biomass and DNA read frequencies^38^?
5. Can we understand the prevalence of “false positive” or “false negative” records^39,40^ based on species traits or characteristics?
6. Do patterns related to all the above questions differ between ants, springtails and termites?

## Material and methods

Additional details for all method sections are provided in Appendix S1.

### Study site and field sampling

**Figure 1:**
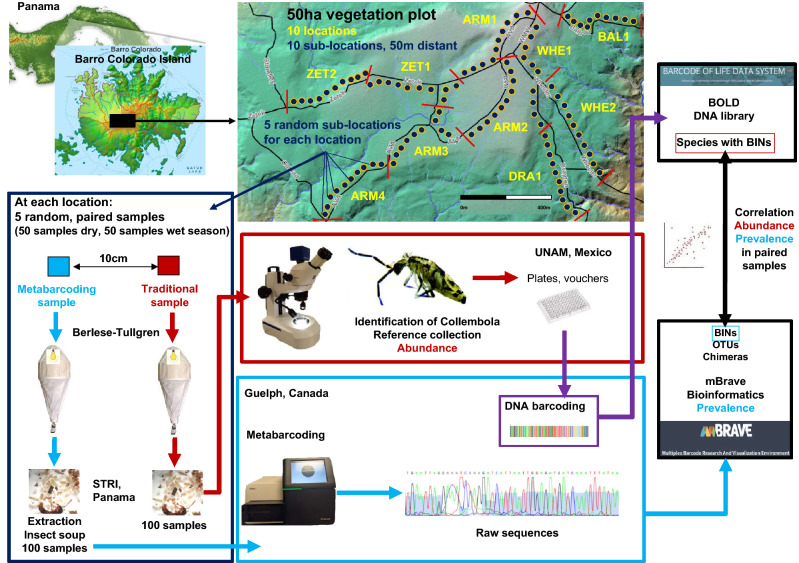
Summary diagram illustrating the workflow of samples for springtails. The black square in the BCI map indicates the location of the 50 ha plot. Traditional samples of ants and termites were processed at STRI in Panama. The Panama and BCI maps are freely available at https://nsf.gov/news/mmg/mmg_disp.jsp?med_id=74874 &from. Map from the 50 ha plot is also freely available at https://stridata-si.opendata.arcgis.com/maps/SI::barro-colorado-island-topographic-webmap/explore?location=9.157000%2C-79.848900%2C14.68.

Assemblages of ants, springtails and termites were surveyed on Barro Colorado Island (9.15° N, 79.85° W; 120–160 masl) in Panama. The 1542 ha Barro Colorado Island is covered with lowland tropical forest and was created around 1910, when the Chagres River was dammed to fill the Panama Canal. Samples were obtained from the 50 ha ForestGEO vegetation dynamics plot, which is described in Ref.^31^. We considered ten locations (500 m sections of trails inside or near the plot) that are used for long-term arthropod monitoring and described in Refs.^33,44^ (Fig. 1, Table 1).

**Table 1:** Number of individuals and species of ants, springtails and termites (with BINs; traditional samples) and number of reads, reads in BINs, reads in OTUs and species (metabarcoding samples) recorded in samples grouped by location.

Location	Taxonomic samples		Metabarcoding samples		Reads in OTUs^c^	No. Spp
	No. ind	No. Spp	Total reads^a^	Reads in BINs^b^		
ARM1	401	38	2,959,781	707,227	736,268	43
ARM2	441	40	3,253,108	646,202	1,073,925	41
ARM3	656	65	3,650,551	929,015	856,161	56
ARM4	774	57	3,337,128	135,814	1,397,542	42
BAL1	274	42	2,804,034	622,318	876,746	38
DRA1	344	39	2,623,464	345,515	934,815	42
WHE1	361	42	2,351,194	627,690	574,774	36
WHE2	63	24	2,424,845	813,851	660,620	49
ZET1	688	48	3,805,815	635,410	1,543,644	42
ZET2	845	58	3,252,451	725,566	1,118,475	32

^a^Total no. of reads uploaded in mBRAVE.

^b^Matched BINs, mostly arthropods.

^c^Arthropods and non-arthropods, the later including vertebrates, invertebrates and plants.

Each location was divided into ten sub-locations (Fig. 1; see statistical methods below and Appendix S2 for explanation of this approach). For each of the ten locations, we randomly selected five sub-locations. At each of these selected sub-locations, we took two paired samples 10 cm distant from each other. We obtained 50 paired soil samples in March 2017, during the dry season. We repeated this sampling protocol in December 2017, during the wet season, with different random samples and obtained 100 paired soil samples for the two seasons. We used a 20 × 20 cm frame (400 cm^2^) to delineate one sample and scoop all litter and soil up to a depth of ca. 5 cm or slightly deeper into a container, calibrating each sample to a final volume of 2 L. We restricted our sampling to the first 5 cm of the litter/soil as tropical soils tend to be shallow, with most of the faunal diversity concentrated in the top few centimeters^45^. To avoid contamination between samples in the field, we used laboratory gloves and disinfected all tools and receptacles with commercial bleach (Clorox de Centroamérica; hypoclorite of sodium 3.5%, hydroxide of sodium 0.3%), after which sampling gear was rinsed with distilled water, to clean bleach residues.

Each pair of samples consisted of two categories: “traditional samples”, from which the soil fauna was extracted and sorted manually according to morphology and molecular data (see below), and “metabarcoding samples” which were analyzed using DNA metabarcoding. Traditional samples were set 4–5 h after collecting in Berlese-Tullgren funnels^46^. Arthropods were extracted during the first 24 h at ambient external temperature (i.e., without heat), and subsequently during the next 48 h with additional heat provided by 25 W bulbs. Berlese-Tullgren extracts were washed, arthropods were separated manually from soil debris and stored in 95% ethanol in a freezer at – 20 °C until further analysis.

Metabarcoding samples were extracted similarly with Berlese-Tullgren with the difference that equipment was thoroughly disinfected with bleach and rinsed with distilled water before and after each sample extraction. Metabarcoding samples were stored at − 20 °C until being analyzed at the Hajibabaei laboratory at the University of Guelph, Canada, which occurred approximately three months after collection. Geographic coordinates of the samples are indicated in Ref.^33^ and Fig. 1 summarizes the workflow of samples.

### Processing of traditional samples

Ants, springtails and termites were all identified via morphological and molecular data (individual-based DNA barcoding). For morphological identification, ants were pinned and assigned to species using the ant reference collection of the ForestGEO Arthropod Initiative at the Smithsonian Tropical Research Institute. Springtails were first cleared in 10% KOH, then in lactophenol, and mounted on microscopic slides in Hoyer’s solution. The slides obtained were identified at the Laboratorio de Ecología y Sistemática de Microartrópodos at the Universidad Nacional Autónoma de México (UNAM) using UNAMs extensive reference collections of springtails and specialized literature. Termites were stored in ethanol and soldiers identified using the termite reference collection of the ForestGEO Arthropod Initiative.

The standard DNA barcode region of the gene cytochrome c oxidase subunit I (COI) was sequenced for a subset of the specimens collected. DNA barcoding using Sanger sequencing was conducted at the Centre for Biodiversity Genomics, University of Guelph, using methods described in Wilson^47^. When possible, we sequenced a maximum of five individuals per species. In total, we generated 324 new COI sequences, corresponding to 328 specimens of 171 ants, 114 springtails and 43 termites. These sequences were added to BCI projects BCIFO, BCICL and BCIIS of the Barcode of Life Data System (BOLD; http://www.barcodinglife.org/index.php), which summed 2,892 sequences (Appendix S1, Supplementary Table S1). These molecular data were used to confirm identifications based on morphology. Each species was attributed a Barcode Index Number (BIN) according to BOLD, which can be used as a proxy taxonomic unit in absence of binomial identification^14^ (e.g., “*Ectatomma* ACH3273 ruidum”).

### DNA metabarcoding

Metabarcoding samples were transferred into separate 50 mL tubes and centrifuged to collect soil and tissue at the bottom of the tube. Excess preservative ethanol was removed, and samples were left in an incubator at 65 °C for 3–6 h to evaporate any residual ethanol. Samples were briefly vortexed to dislodge and coarsely homogenize the dried pellet before transferring ~ 0.25 g to a PowerSoil bead tube with sterile forceps. Whole DNA was extracted using DNeasy PowerSoil Kit (Qiagen: Toronto, ON, Canada) according to protocol, eluting with 30 µL, buffer C5. Isolated DNA was amplified through a two-stage Polymerase Chain Reaction (PCR) for two overlapping amplicons from the DNA barcode region (Helix I to Helix V) of the COI gene. These amplicons were: (1) BR5: size ca. 310 bp; forward primer B: 5′CCIGAYATRGCITTYCCICG^23^; reverse primer ArR5: 5′GTRATIGCICCIGCIARIACIGG^24^. (2) F230R: size ca. 230 bp: forward primer LCO1490: 5′-GGTCAACAAATCATAAAGATATTGG^48^; reverse primer 230_R: 5′ CTTATRTTRTTTATICGIGGRAAIGC^49^. For the map of primers and amplicons see Ref.^50^. These were selected as proven to be effective to optimize recovery of species and genus richness in tropical arthropods^22,50^. Reactions had a standard mix as indicated in Appendix S1, for a total of 25 μL per reaction. A negative control (i.e., reaction with 2 μL water instead of DNA) was included to ensure PCR reagents were free of contamination. Reactions underwent 35 cycles of 94 °C for 40 s, 46 °C for 60 s, 72 °C for 30 s using an Eppendorf Mastercycler ep gradient S thermocycler. PCR amplification was visually confirmed through gel electrophoresis using a 1.5% agarose gel. PCR products were purified following the MinElute PCR Purification kit (Qiagen; Toronto, Ontario, Canada) standard protocol, eluting with 15 μL molecular biology grade water. A second round of PCR with primers that included the Illumina adapter sequence was run under the same conditions, using the purified product from the first round of PCR as template. Two PCRs with 35 cycles are sufficient for providing consistent and robust results for many samples across different sample types. This protocol has been used routinely with consistent results for biodiversity, ecological and biomonitoring studies (e.g., Refs.^22,49,51^). Additionally, the high number of PCR cycles in the second round was practical because amplification was inadequate at lower cycles, and some samples failed. Purified second round PCR product was sequenced on an Illumina MiSeq using the v3 MiSeq sequencing kit (300 bp × 2).

### Bioinformatics

The bioinformatics of metabarcoding remains in flux^27^. A wide array of metabarcoding tools exists, and their choice (and relevant parameters) is essential for meaningful results^52^. Available tools include, for example, Mothur^53^, Qiime^54^ or Obitools^55^. However, an important restriction that pertains to all these tools is the use of customized databases for taxonomy classification. We relied on the mBRAVE cloud-based platform (“Multiplex Barcode Research and Visualization Environment”; http://www.mbrave.net/^56^) since it seamlessly integrates BOLD data as reference datasets, which for BCI represent the best curated COI sequence datasets available. mBRAVE employs a typical workflow based on mature bioinformatic methods and is comparable to other available bioinformatics tools. The sequence reads resulting from metabarcoding were uploaded into the project MBR-BCISOIL of mBRAVE. mBRAVE parameters were optimized for classifying sequences with BOLD datasets which have already been curated and the various sequence errors removed. Hence, the stringent read processing was a bit more relaxed. All mBRAVE parameters are indicated in Supplementary Table S1, but the workflow can be summarized as follows.

The paired ends of the two overlapping amplicons were pooled (i.e., treated as one fastq file without any merging) as opposed to merging, since (a) we were expecting very high matches to mBRAVE datasets (see below) with variation within a BIN being small; and (b) a sensitivity analysis, where we varied most mBRAVE parameters one by one to optimize the greatest number of BINs recovered in mBRAVE datasets, indicated an optimal recovery of BINs with pooled sequences (Supplementary Appendix S1 and Supplementary Table S2). Reads were trimmed at each end (25 bp) to remove primer and adapter sequence, then filtered for a minimum quality score of QV20 and for a minimum and maximum size of 100 bp and 500 bp, respectively (Appendix S1). Denoising, dereplication and removal of chimeric reads, were performed with VSEARCH^57^, integrated into the mBRAVE pipeline (Appendix S1). Erroneous sequences were detected by a divergence range from reference sequences, with parameters as indicated in Supplementary Table S1. Sequences were identified to BIN level by matching them to reference BINs based on BOLD library datasets tailored for this study, using VSEARCH. These datasets included sequences obtained specifically for this study (2571 BINs for BCI arthropods) and publicly available datasets within BOLD (Appendix S1, Supplementary Table S1). BINs assignments were realized at 3%, a commonly employed threshold^58^. Reads without a match to reference BOLD datasets were clustered into operational taxonomic units (OTU) based on (1) the minimum OTU size (n = 1 read); (2) OTU threshold (maximum distance inside a generated OTU, a conservative 2%); and (3) exclusion from the OTU threshold when sequencing error introduces spurious haplotypes (chimeras and/or sequence errors, 3%). The BOLD datasets that we used for analyses are currently the best ones available for ants, springtails and termites from BCI (Supplementary Appendix S1 and Supplementary Table S1). It is important to note that, although sequence merging is often recommended to remove potentially erroneous sequences, we opted to pool our reads without merging as this retrieved a higher number of BINs. This strategy is possible because (a) we count with an extensive barcode reference library which has been continuously updated and curated by co-authors of this manuscript and represents the most complete insect barcode reference library available for Barro Colorado Island; and (b) this represents a first step towards a standardized and automated arthropod monitoring protocol where our primary goal is to detect reads matching known species barcodes.

Depending on the data and aims of the metabarcoding study, this approach may or may not be relevant.

### Statistical analyses

To ensure sound comparisons between traditional and metabarcoding samples, we removed five pairs of samples for which DNA metabarcoding samples failed (all from the dry season; PCR failed), leaving 95 pairs of samples for analysis. For monitoring, it is necessary to identify unequivocally species by their binomials or BINs, so that they can be cross-referenced among years. Hence, for species sorted manually, we only considered them if they had been sequenced and had a BIN, given that some species failed to be sequenced (see “Results”). Common (as opposed to rare) species were defined as species belonging to the first quartile of the species-rank prevalence distribution^59^, for species sorted in traditional samples. The 95 metabarcoding samples resulted in a total of 122 sequencing runs (i.e., single fastq files produced by the sequencer through demultiplexing or from a single sample) and hence sampling effort was not strictly identical among locations sampled. However, unequal (but similar) sampling effort among locations did not affect any of our questions, and small differences in sample size among locations were ignored.

Despite harvesting traditional and metabarcoding samples only 10 cm apart, we can still expect discrepancies in species abundance and faunal comparison between these samples. This results from soil organisms, such as ants, to be very patchily distributed when studied at the 1 m^2^ scale^60^. To reduce this effect, our analyses emphasized data in which samples were grouped per location, as to evaluate differences at a higher scale. This issue is further discussed in Appendix S2.

### Question1: similarity between traditional and metabarcoding samples

To compare species richness between sample types (traditional and metabarcoding) and taxa (prevalence data), we computed rarefaction curves and estimates of total species richness with the R package iNEXT^61^. To visualize the differences in faunal composition between traditional and metabarcoding samples, we generated differential heat trees using the Metacoder R package^62^. These trees help identifying the taxonomic hierarchy with terminal nodes representing either BINs or binomial species. With a prevalence matrix, we used the function ‘compare_groups’ of Metacoder and the resulting mean difference between treatments to plot the differential heat tree. The differences, however, were formally tested using a Procrustes rotation, which rotates a configuration (for example ordination results) to maximum similarity with another configuration. First, for each dataset (traditional and metabarcoding), we pooled samples by the 10 locations at our study site (Table 1; Fig. 1) and considered matrices of species × locations, filled with the number of samples in which the species was recorded for each location (prevalence; maximum possible of 10 samples for each location). We then performed a non-metric multidimensional scaling (NMDS) for each of the traditional and metabarcoding matrices with the function ‘metaMDS’ of the R package vegan, followed by a Procrustes rotation of the two ordinations with the function ‘procrustes’ of the same package^63^. The function ‘protest’ of vegan was used to tests for non-randomness (‘significance’) between the traditional and metabarcoding NMDS (999 permutations). We performed Procrustes rotations separately for ants, springtails and termite data, and either for all species surveyed or restricted to common species.

### Question 2: regressions between abundance and prevalence

We considered the relationships between the following variables, for each species: (a) total abundance vs. prevalence in traditional samples; (b) total abundance in traditional samples vs. prevalence in metabarcoding samples; and (c) prevalence in traditional samples vs. prevalence in metabarcoding samples. We tested these three relationships for the three taxonomic groups together, and then for each group separately, considering either all species or only common species. We first checked linear and non-linear relationships with CurveExpert Professional^64^, considering the highest coefficient of determination (R^2^) and the lowest Akaike information criterion corrected (AICc) of all models. Given that linear relationships provided the best fit for all models tested, we fitted ordinary least squares regressions to all tested relationships.

### Question 3: seasonal shifts

We first calculated, for each species collected both in the dry and wet seasons (n = 62), the difference in the prevalence in the wet season samples minus the prevalence in the dry season samples, separately for traditional and metabarcoding samples. We then fitted ordinary least squares regressions between the seasonal difference in traditional samples and that in metabarcoding samples. We performed these regressions for all species, ants, springtails and termites, but not separately for common species, because of low sample size. Eventually, to evaluate the similarity between the traditional and metabarcoding samples regarding shifts in faunal composition between the dry and wet season, we considered separately for traditional and metabarcoding samples, a prevalence matrix of species (n = 62) x season (dry or wet). We then calculated the correlation between the two matrices using a Mantel test, computed with the function ‘mantel’ of the R package vegan (10,000 permutations^63^).

### Question 4: regressions between biomass and read frequency

We considered only ants, for which we had reliable measurements of different variables accounting for body size, as well as an allometric correlation of body length to body weight. Analyses were restricted to ant species present in metabarcoding samples for which we had good measurements (1–5 individuals measured per species, n = 28 species). We tested linear and non-linear relationships with CurveExpert Professional^64^, including, for each species, as dependent variables the total number of reads, the relative read abundance^65^, the total number of sequences and prevalence in metabarcoding samples, and as independent variables either body length (mm), pronotum width (mm), Weber’s length (mm) or body weight (g), measured from ant workers in collections of the ForestGEO Arthropod Initiative.

### Question 5: false positive and negative records

Assuming no taxonomic errors, we considered all species occurring in metabarcoding samples but not in traditional samples as “possible false positive”, those occurring in traditional samples but not in metabarcoding samples as “possible false negative” and the rest of species as “well surveyed”. Furthermore, we considered species as “probable false positive” and “probable false negative” when their prevalence in samples was ≥ 10, in metabarcoding samples and traditional samples, respectively (i.e., when at least one specimen was collected potentially at each of the locations). We performed a discriminant analysis to test whether morphological traits such as body length, pronotum width and Weber’s length (Question 4) delineated some of the categories defined above. This analysis concerned only Formicidae and the species in categories “probable false negative” and “well surveyed”, as sample size was too low for species in other categories (morphological measurements lacked for most of false positives). Discriminant analysis was performed with the function ‘discrim’ of Systat version 13.1 (Systat Software, Inc., Chicago, IL). Given that the barcodes for our false negative samples exist, we used the ecoPCR^66^ program to run an in silico PCR to test whether or not our primers could amplify these regions. Barcodes were downloaded from the NCBI database to create a reference dataset to match with our PCR primer pairs allowing up to 15 mismatches between primer and target sequences. To test for differences between taxa (Question 6), whenever possible we kept analyses separate among taxa when investigating Questions 1–5.

## Results

Traditional samples yielded 1970, 5037 and 226 individuals classified into 80, 111 and 15 morphological species of ants, springtails and termites, respectively. Metabarcoding samples produced 30.46 million reads, including 15.96 million post filter reads (mean sequence length = 233 bp). Most of the reads representing BINs included arthropods and more than half of these BINs were assigned to the focal taxa studied (Supplementary Table S3). We validated 396 BINs of arthropods (Supplementary Tables S4, S5) from our library datasets. Diptera, Hymenoptera, Coleoptera, Collembola and Isoptera were best represented in terms of species richness (unique BINs, Supplementary Table S5). Doubtlessly, total species richness in these samples may be higher as most of arthropod species from BCI have not been barcoded yet and, hence, could not be validated with our library databases. The validation of the remaining OTUs not assigned to BINs is challenging, as many may be artefactual^67^. It is beyond the scope of the present contribution to discuss this issue, although we identify complex of species in our focal taxa that may be worth investigating in the future (Supplementary Appendix S2 and Supplementary Table S6). Given the low resolution of OTU data, we now focus on the detection of validated BINs detected in metabarcoding samples for our focal taxa, which included 49, 37 and 34 BINs for ants, springtails and termites, respectively. About 98%, 37% and 100% of species of ants, springtails and termites, respectively, observed in traditional samples had BINs (DNA extraction failed for some species; full list in Ref.^33^), and could hence be directly compared with metabarcoding samples. In sum, our study system consisted of 101, 52 and 39 species with BINs (hereafter “species”) of ants, springtails and termites, respectively, that were amenable to comparisons between traditional and metabarcoding samples (Supplementary Fig. S1). Table 1 summarizes the number of individuals, species and reads per location.

### Question 1: similarity between traditional and metabarcoding samples

**Figure 2:**
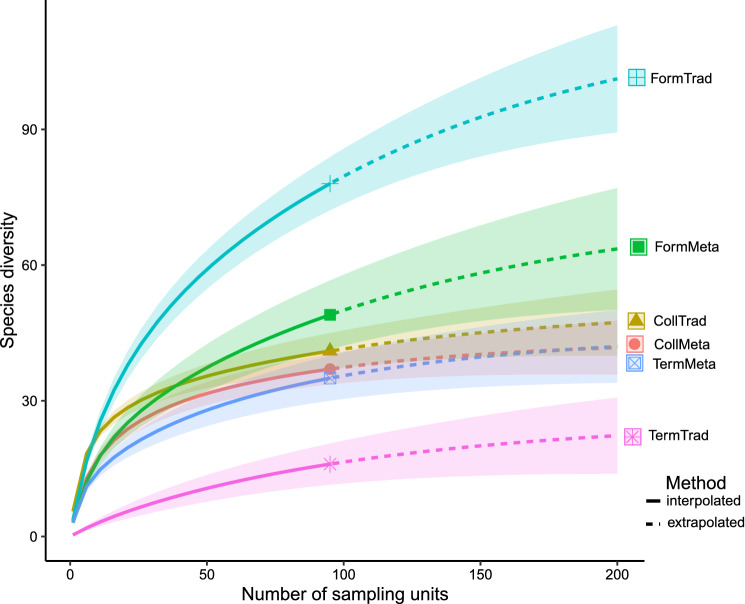
Accumulation of species richness vs. the number of samples for ants, springtails and termites, detailed for traditional and metabarcoding samples. CollMeta, CollTrad = Collembola in metabarcoding and traditional samples; FormMeta, FormTrad = Formicidae in metabarcoding and traditional samples; TermMeta, TermTrad = termites in metabarcoding and traditional samples. The graphic was created with iNext 2.0.20 https://cran.r-project.org/web/packages/iNEXT/index.html^61^.

Observed species richness (95 samples), extrapolated species richness (200 samples) and total estimated species richness was higher in traditional samples than in metabarcoding samples for ants (Fig. 2; traditional samples: 78 species observed, 117.0 ± 18.6 (s.e.m.) spp. estimated, metabarcoding samples: 49 spp., 73.7 ± 14.9 spp.). In contrast, total species richness in springtails was rather similar between traditional and metabarcoding samples (with 41 vs. 37 species observed, respectively; 51.0 ± 8.9 vs. 43.3 ± 5.9 species estimated), while for termites, it was lower in traditional samples than in metabarcoding samples (15 vs. 34 species observed, respectively, 26.0 ± 8.9 vs. 45.0 ± 7.9; Fig. 2). These patterns were broadly similar when we restricted the data to common species, suggesting also that the number of common species discovered by twice the number of metabarcoding samples would not be much different than currently detected in ca. 100 samples (Supplementary Fig. S2; for the identity of common species, see Question 2).

**Figure 3:**
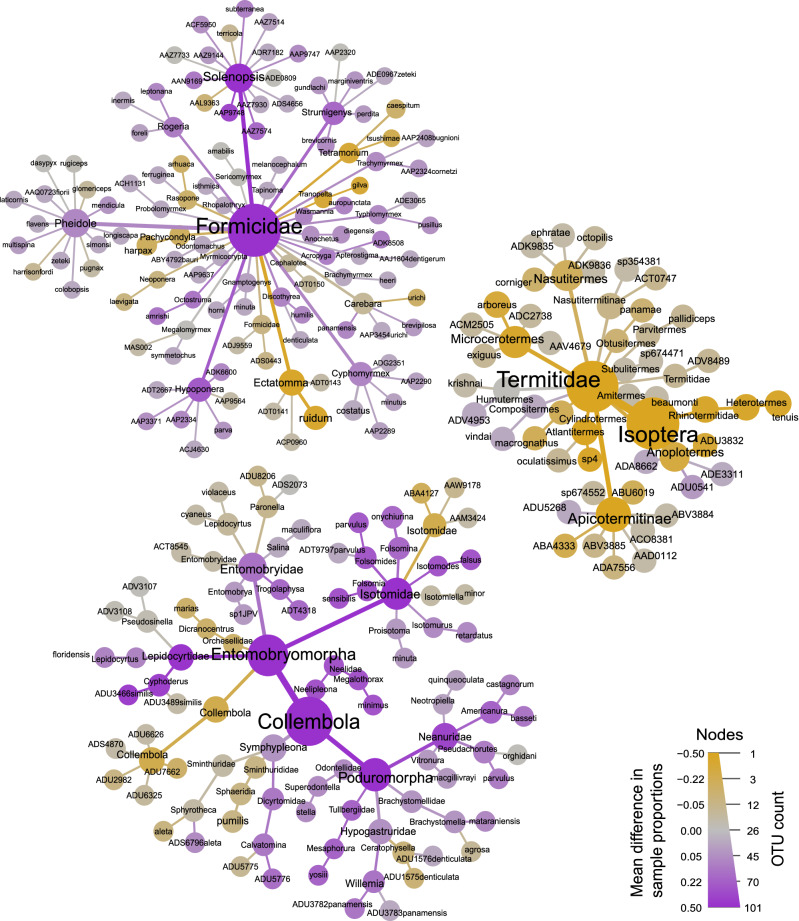
Taxonomic comparison between identification methods for the three focal groups. The size of each node represents the number of BINs for each hierarchical classification, while differences in color correspond to sampling methods (purple = traditional samples; brown-orange = metabarcoding samples; gray = occurring in both). The heat map was created with Metacoder 0.3.5 https://cran.r-project.org/web/packages/metacoder/index.html^62^.

The heat trees visualizing differences in the number of BINs detected in traditional and metabarcoding samples indicated that termites were better surveyed with metabarcoding samples (Fig. 3). When considering all species surveyed, the Procrustes rotations performed with the NMDS including traditional and metabarcoding data for ants, springtails and termites were not significantly correlated (Supplementary Fig. S3; all correlations with p > 0.05; statistics detailed in the figure). These patterns were similar when we restricted the analyses to the 34 most common species, except for common termite species, whose two ordinations were significantly correlated (Supplementary Fig. S3). In this case the information provided by the Procrustes rotation was rather low, as this concerned only four species. Note that after removal of the outlier location “WHE2” in Collembola (Supplementary Fig. S3), the Procrustes rotations for both all and common species were still not significantly correlated (all species: n = 52 species considered, m12 = 0.785, r = 0.46, *p* = 0.34; common species: n = 10, m12 = 0.713, r = 0.54, *p* = 0.19).

### Question 2: correlation between abundance and prevalence

**Figure 4:**
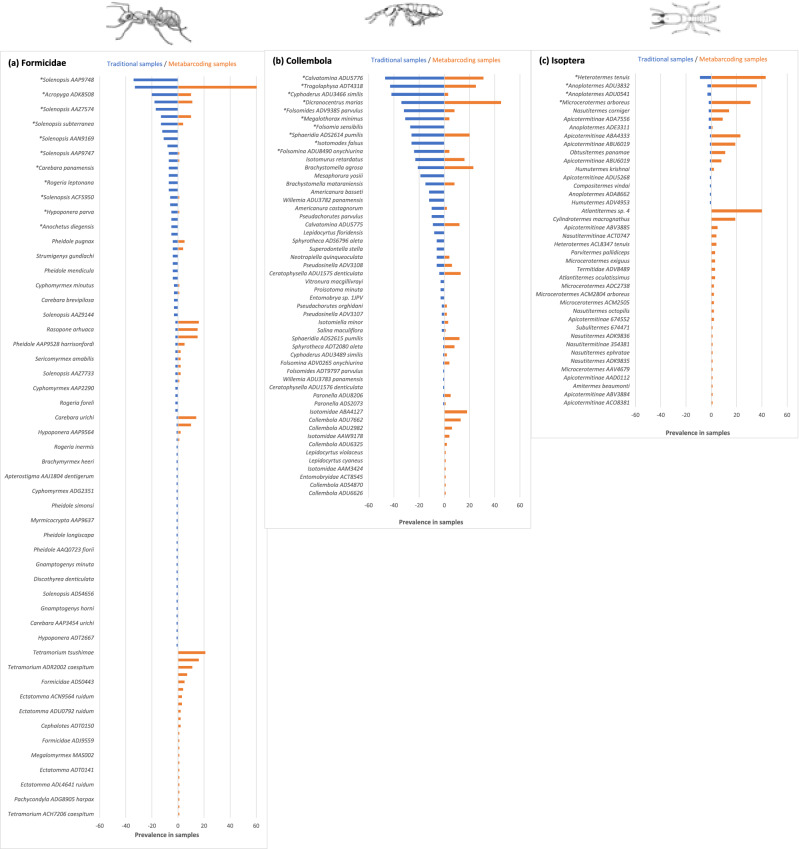
Species ranked by, first, prevalence in traditional samples (blue bars) and, second, prevalence in metabarcoding samples (orange bars) for (**a**) Formicidae, (**b**) Collembola and (**c**) Isoptera. Species indicated by “*” represent common species as defined in this study.

There were strong positive relationships between total abundance and prevalence in traditional samples when all species were analyzed together (67% of variance explained), as well as for ant (69%) and springtail species (72%) analyzed on their own, but not for termite species (4%; Table 2). The relationship between abundance in traditional samples and prevalence in metabarcoding samples was weaker. The strongest positive relationship existed for all ant species (24% of variance explained), followed by all springtail species (16%). This relationship was not significant for termite species. Restricting the data sets to common species did not notably improve the fit of relationships (Table 2). There were also significant but relatively weak correlations between the prevalence of species in traditional and metabarcoding samples. The highest proportion of variance explained was observed for termites (35%), springtails (29%) and ants (19%). Data sets restricted to common species did not improve the relationships, with the notable exception of Formicidae (40% of variance explained; Table 2). Figure 4 summarizes visually the prevalence of species in traditional and metabarcoding samples.

**Table 2:** Linear regressions including Formicidae, Collembola, Isoptera species and the following independent and dependent variables: *AbTaxo* abundance in traditional samples, *PrTaxo* prevalence in traditional samples, *PrMeta* prevalence in metabarcoding samples, *PrTaxo* prevalence in traditional samples. Regressions for common species of termites could not be tested because of small sample size.

Group	Independent	Dependent	n	R^2^	Constant (s.e.)	Coefficient (s.e.)	F	*p*
All species	AbTaxo	PrTaxo	134	0.676	2.408 (0.548)	0.120 (0.007)	275.4	< 0.001
All species	AbTaxo	PrMeta	134	0.139	2.853 (0.922)	0.056 (0.012)	21.2	< 0.001
All species	PrTaxo	PrMeta	193	0.144	2.803 (0.695)	0.400 (0.070)	32.2	< 0.001
All common spp.	AbTaxo	PrTaxo	34	0.491	8.076 (2.349)	0.093 (0.017)	30.8	< 0.001
All common spp.	AbTaxo	PrMeta	34	0.08	n.s	n.s	2.8	0.106
All common spp.	PrTaxo	PrMeta	34	0.144	1.633 (4.368)	0.465 (0.201)	5.6	0.027
Formicidae	AbTaxo	PrTaxo	78	0.694	2.372 (0.428)	0.084 (0.006)	172.3	< 0.001
Formicidae	AbTaxo	PrMeta	78	0.240	0.974 (0.876)	0.064 (0.013)	24.0	< 0.001
Formicidae	PrTaxo	PrMeta	101	0.190	0.902 (0.796)	0.568 (0.118)	23.3	< 0.001
Formicidae common spp.	AbTaxo	PrTaxo	20	0.617	6.904 (1.558)	0.064 (0.012)	29.0	< 0.001
Formicidae common spp.	AbTaxo	PrMeta	20	0.271	− 0.779 (3.663)	0.073 (0.028)	7.0	0.019
Formicidae common spp.	PrTaxo	PrMeta	20	0.398	− 7.919 (4.592)	1.076 (0.312)	11.9	0.003
Collembola	AbTaxo	PrTaxo	41	0.717	3.170 (1.492)	0.149 (0.015)	99.0	< 0.001
Collembola	AbTaxo	PrMeta	41	0.156	3.153 (1.887)	0.051 (0.019)	7.2	0.011
Collembola	PrTaxo	PrMeta	52	0.289	2.218 (1.371)	0.376 (0.084)	20.3	< 0.001
Collembola common spp.	AbTaxo	PrTaxo	10	0	n.s	n.s	0.0	0.965
Collembola common spp.	AbTaxo	PrMeta	10	0.026	n.s	n.s	0.2	0.659
Collembola common spp.	PrTaxo	PrMeta	10	0.223	n.s	n.s	2.3	0.168
Isoptera	AbTaxo	PrTaxo	15	0.039	n.s	n.s	0.5	0.48
Isoptera	AbTaxo	PrMeta	15	0.035	n.s	n.s	0.5	0.507
Isoptera	PrTaxo	PrMeta	40	0.351	4.052 (1.686)	4.341 (0.957)	20.6	< 0.001

### Question 3: seasonal shifts

**Figure 5:**
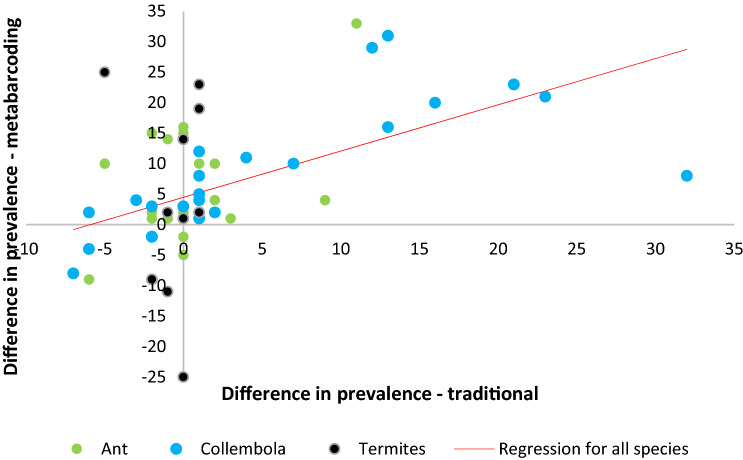
Plot of the difference in species’ prevalence between wet and dry season in traditional samples vs. that in metabarcoding samples.

The regression for all species between the seasonal difference in prevalence in traditional samples and that in metabarcoding samples was significant, and explained 26% of variance (F_1,60_ = 20.5, *p* < 0.001; Fig. 5). This regression was strong for springtail species (F_1,24_ = 26.2, p < 0.001, R^2^ = 0.523), was significant for ant species (F_1,24_ = 6.6, *p* = 0.017, R^2^ = 0.215), but not for termite species (F_1,8_ = 0.09, *p* = 0.76, R^2^ = 0.012). The Mantel test indicated that the matrix of species × seasons of traditional samples was significantly correlated with that of metabarcoding samples (all species considered; r = 0.336, *p* = 0.003).

### Question 4: correlation between biomass and read frequency

For the 28 ant species amenable to analyses we observed in metabarcoding samples strong but expected linear relationships between the number of reads for one species and the number of sequences representing its BIN (R^2^ = 0.825, F_1,26_ = 122.6, *p* < 0.001), as well as between the number of reads and the species prevalence (R^2^ = 0.964, F_1,26_ = 703.1, *p* < 0.001). There was a weak positive linear relationship between estimated body weight and read frequency (R^2^ = 0.142, F_1,26_ = 4.3, *p* = 0.048). Similarly, there was a weak logistic (sigmoid) relation between body weight and relative read abundance (y = a/(1 + b × e^(−cx)^); R^2^ = 0.141, F_1,26_ = 4.2, *p* = 0.048). However, there was no linear relationship between body weight and prevalence in metabarcoding samples (R^2^ = 0.054, F_1,26_ = 1.5, *p* = 0.235).

### Question 5: false positive and negative records

Out of 192 species in total, 58 species were classified as possible false positive (30% of the total) and 72 species as possible false negative (37%; Fig. 4). This was expected a priori since the proportion of rare species is always high in tropical insect surveys^68^ (32.6% of species collected/detected as singleton in either traditional or metabarcoding samples in this study). More revealing, “probable” false positive and negative species represented 14.0% and 20.0% of the number of species that occurred in 10 or more samples (n = 50), and 12.5% and 18.8% of the total number of common species, respectively. Probable false positives included three ant, two termite and two springtail species, whereas probable false negatives included six springtail and four ant species (Table 3). The COI barcodes of all of these false negative species proved to be amplifiable using our primers in our in silico evaluation (Supplementary Table S7).

**Table 3:** List of species considered as "probable false positive" (i.e., high prevalence in metabarcoding samples but absent in traditional samples) and as "probable false negative" (i.e., high prevalence in traditional samples but absent in metabarcoding samples). *Prev* Prevalence in samples, *Rec* no. of records on BCI, years 2008–2019, *Spm* no. of specimens with BINs. Sources: BOLD (https://www.boldsystems.org/); Termite catalog (http://164.41.140.9/catal/); antwiki (https://www.antwiki.org).

Species	Family	BIN	Prev	BCI	Distribution	Notes
**(a) Probable false positive**						
*Atlantitermes* sp. 4	Termitidae	TAX:674460	40	No	Ecuador, Peru	*Atlantitermes* spp. Neotropical; *A. kirby* on BCI
*Tetramorium tsushimae*	Formicidae	BOLD:ACV4760	21	No	Japan, Korea, China	*Tetramorium bicarinatum* on BCI (BIN BOLD:AAA5578)
*Cylindrotermes macrognathus*	Termitidae	BOLD:AAP9583	19	Yes	Costa Rica, Panama	156 specimens collected on BCI
Isotomidae ABA4127	Isotomidae	BOLD:ABA4127	18	No	Africa, Indonesia	Information minimal
*Ectatomma* ACH3273 ruidum	Formicidae	BOLD:ACH3273	16	Yes	Neotropical	Four cryptic species of "*E. ruidum*" are present on BCI
Collembola ADU7662	Entomobryidae?	BOLD:ADU7662	13	Yes	Panama	44 specimens collected from Malaise trap on BCI
*Tetramorium* ADR2002 caespitum	Formicidae	BOLD:ADR2002	11	No	China	*Tetramorium bicarinatum* on BCI (BIN BOLD:AAA5578)
**(b) Probable false negative**						
*Solenopsis* AAP9748	Formicidae	BOLD:AAP9748	34	Yes	Panama	Rec 1008, Spm 12
*Folsomia sensibilis*	Isotomidae	BOLD:ADS5886	27	Yes	Panama	Rec 41, Spm 1
*Isotomodes falsus*	Isotomidae	BOLD:ADT5082	26	Yes	Panama	Rec 32, Spm 1
*Mesaphorura yosiii*	Tullbergiidae	BOLD:ADS1278	19	Yes	Panama	Rec 26, Spm 2
*Solenopsis* AAZ7574	Formicidae	BOLD:AAZ7574	17	Yes	Panama	Rec 109, Spm11
*Octostruma amrishi*	Formicidae	BOLD:ABX5315^a^	12	Yes	Panama	Rec 630, Spm 19; two BINs: ABX5315/AAP3374
*Willemia* ADU3782 panamensis	Hypogastruridae	BOLD:ADU3782	12	Yes	Panama	Rec 17, Spm 1; one close species with BIN ADU3783 on BCI
*Americanura basseti*	Neanuridae	BOLD:ADS4642	12	Yes	Panama	Rec 16, Spm 3
*Solenopsis* AAN9169	Formicidae	BOLD:AAN9169	11	Yes	Neotropical	Rec 560, Spm 19
*Pseudachorutes parvulus*	Neanuridae	BOLD:ADV9385^a^	10	Yes	Panama	Rec 16, Spm 5; two BINs: ADT9797/ADV9385

^a^See notes.

For ants, the discriminant analysis delineating the possible false negative (n = 28) and the well surveyed species (n = 25) was overall significant (Wilks's Lambda = 0.844, *p* = 0.039), with Weber’s length and body length as loading most on the first discriminant axis (eigenvalue = 0.185; canonical correlation = 0.395). The trend was for possible false negative species to have lower values of Weber’s and body length in comparison with well surveyed species.

## Discussion

The development of molecular methods allows for greater opportunities to monitor the lesser-known biodiversity, including arthropods from tropical soils. Previous metabarcoding studies have discussed topics such as profiling communities, beta diversity, dispersal, community assembly and body size^18–22^. However, few studies have been considering practical questions related to monitoring over time and the elaboration of time series. The current project represents one of the first attempts to develop protocols for monitoring in the long term the soil fauna in the tropics.

After considering 95 spatially paired samples processed either traditionally or with metabarcoding, representing a total of 101, 52 and 40 species of ants, springtails and termites, respectively, we provide a list of positive items for developing a DNA metabarcoding protocol for the monitoring of tropical soil arthropods. Termites were better surveyed by metabarcoding than by traditional samples and springtails equally well. Doubling the number of samples would have not resulted in the detection of many more species. There was a positive correlation between the abundance of ant and springtail species in traditional samples and their prevalence in metabarcoding, although it was relatively weak (12–27% of variance explained). Seasonal shifts in species prevalence between the dry and wet season were overall correlated between traditional and metabarcoding samples. Further, changes in faunal composition between dry and wet season were also correlated between traditional and metabarcoding samples. Probable false positive species represented 14.0% of the number of species that occurred in 10 or more samples. Close scrutiny of these results indicated that 3 species could be classified as true errors (see below), lowering the proportion of probable false positive species to 6.3%.

Conversely, the following items appear more challenging for developing a comprehensive monitoring protocol. The overall species richness of metabarcoding samples was underestimated in comparison to traditional samples. This effect was mostly due to ants, emphasizing the selectivity of metabarcoding for particular taxa. This pattern also prevailed when we restricted the analyses to common species. This may result from different factors, including selective PCR primer amplification bias^50^, better efficiency of nuclear DNA over mitochondrial DNA to delineate species in ants^69^, amplification bias in AT rich Hymenopteran genomes^70^ or extensive heteroplasmy in the mitonchondrial DNA of certain species complex, such as *Ectactomma ruidum*^71^. The faunal composition of metabarcoding samples was significantly different from that in traditional samples, and this pattern was similar when we restricted the data to common species. There was no correlation between the abundance of termite species in traditional samples and their prevalence in metabarcoding samples. Although there was a weak correlation between body weight and relative read abundance, no relation existed between body weight and prevalence in metabarcoding samples. This suggests that species’ body weight may not be useful for refining estimates of species prevalence in metabarcoding samples. Probable false negative species represented 20.0% of the number of species that occurred in 10 or more samples. They included four ant species with high abundance in traditional samples, and that are well collected on BCI with different protocols (see below). It is difficult to interpret these results, but for ants the discriminant analysis suggests that false negative species tended to be smaller than well surveyed ant species. Patterns were often different between the three focal taxa, which challenges the possibility to develop a unique protocol that may be sound for a variety of taxa. Appendix S2 details some methodological considerations emphasizing possible limitations in our study.

Thus, the list of positive findings motivates us to continue tackling the remaining challenges to optimize a monitoring protocol. We should now return to our specific questions as targeting key criteria for an efficient, unbiased, and reliable monitoring protocol.

### Differences between traditional and metabarcoding samples

Regarding our first question, metabarcoding appears to be rather selective at recording certain taxa and species. For example, the proportion of ant species which are overall common (first quartile of the distribution) in litter samples on BCI (data from the ForestGEO Arthropod Initiative, 2008–2019) and which were also recorded in our metabarcoding samples was 43%, whereas the corresponding figure for termites was 75%. From the viewpoint of the field researcher, metabarcoding represents yet another “sampling method” with inherent biases, but with the obvious benefit that time and expertise needed for handling and sorting samples is greatly reduced. Apart from the sensitivity of PCR primers, the reasons for the selectivity observed at the species level are not well understood^72^. Development of better, more general primers (or more specific primers to target focal taxa) could improve current assessments^50,73^. This is not necessarily detrimental to long-term monitoring in soil organisms with metabarcoding, but the limitations of metabarcoding should be acknowledged.

Regarding our second question, there was a correlation between prevalence in metabarcoding samples and abundance in traditional samples, but the proportion of explained variance was relatively low. A partial explanation may be, in the first place, the biases inherent in metabarcoding workflow, as pointed out above. Nonetheless, while similarity in faunal composition between traditional and metabarcoding samples was rather low, our approach to Question 3 showed that the methods is sensitive enough to reveal a good correspondence in the seasonal shifts of species in traditional and metabarcoding samples. This issue is relevant to long-term monitoring by indicating that metabarcoding is sufficiently sensitive to replicate ecological patterns through time in the soil fauna. This pattern has been demonstrated in multi-year analysis of other systems such as macroinvertebrates from wetlands^51^. Hence, related to Questions 2 and 3, we tentatively conclude that it may be possible to use prevalence in metabarcoding samples to derive an estimate of, for example, the annual rate of change in the abundance of species that are well recorded (common) in metabarcoding samples.

### Biomass and read frequencies

With respect to Question 4, we observed that the most common species of ants with high total number of reads are soil nesting species that are common on BCI. However, the relation between ant’s body weight and read frequency or prevalence in metabarcoding samples was either weak or non-existent. For social insects, colony size may be more relevant than worker size or biomass, but we did not have pertinent data to test this relationship. While good correlation between read frequencies and species biomass has been reported for nematodes^74^, this relationship is less clear-cut, weak or absent for arthropods^24,25,27,72,75,76^. Hence, with the current technology available to us, biomass data are unlikely to refine estimation of species prevalence (or biomass) among metabarcoding samples. Bista et al.^76^ showed that shotgun mitogenomic sequencing provides better relationships between reads and biomass than metabarcoding, a finding later confirmed and developed by Ji et al.^77^. Hence, PCR-free methods may potentially offer an alternative avenue towards improved quantification.

### False positives and negatives

The prevalence of false positive species is always a risk, because of contamination or errors during PCR and sequencing^40^. For long-term monitoring this is not an acute problem, as monitoring is often restricted to common species well amenable to statistical analysis^44^. “Probable false positives” as defined in our study can also sometimes be ruled out with a priori knowledge of the local fauna. In evidence of this, *Ectatomma* ACH3273 ruidum and *Cylindrotermes macrognathus* Snyder, 1929 were most likely missed in traditional samples, being part of a complex of cryptic species that can only be delineated with BINs (*Ectatomma* spp*.*) or difficult identification (*C. macrognathus*). The prevalence of *Atlantitermes* sp. 4 on BCI and of Collembola ADU7662 may be plausible, but the other three species, including the two *Tetramorium* which are invasive and urban pests from Asia^78,79^, are most likely errors (Table 3).

Explanations for the prevalence of “probable false negative species” are challenging. These species were reasonably well collected on BCI by the ForestGEO Arthropod Initiative during the period 2008–2019, and they all have BINs included in the reference libraries used. Thus, their absence in metabarcoding samples is unlikely to be due to local scarcity. The prevalence of probable false negative species is more problematic for long-term monitoring^23^. These errors may be generated by primer biases^39^, but this appears less likely in our study, because metabarcoding samples recorded congeneric species well. Further, the in silico evaluation revealed the primers to be capable of amplifying the COI barcodes of the probable false negative species. Another source of error may occur when genuine metabarcodes are assigned to an incorrect taxon due to incomplete or inappropriate reference databases^39^, or to the use of inefficient assignment methods^41^. In our study, false negatives may result mostly from inappropriate referencing of metabarcodes, particularly for species complexes. To reduce the number of false negatives, Ficetola et al.^40^ advised to perform multiple PCR extractions and to use species occupancy models to refine the results. Unfortunately, the occupancy probability of false negative species cannot be estimated by such models, since these species were never encountered in our metabarcoding samples.

## Conclusions

Targeting Question 6, we observed different patterns in the distribution of ants, springtails, and termites in traditional and metabarcoding samples. While Berlese-Tullgren is an efficient method for extracting soil springtails, termites are best surveyed with transects^80^, and ants with Winkler extraction^34^. Thus, the efficiency of metabarcoding may ultimately be improved by choosing a more selective sampling method appropriate for each target taxa.

Our study indicates that metabarcoding of samples extracted with Berlese-Tullgren may be well suited for the long-term monitoring of springtails and termites in tropical rainforests, provided that baseline data, including DNA reference libraries, exist^43^. Vouchered reference libraries are indispensable in this context, as they allow compilation of species traits and delineation of functional groups for a sound interpretation of time-series^32^. For ants, this issue is less clear-cut because many species lacked in metabarcoding samples. Technological improvements that may provide longer sequences and improved detection in reference to DNA libraries may improve ant detection. Irrespective of this issue, monitoring ants with metabarcoding may be possible for certain species with samples obtained from Berlese-Tullgren, and this may be complemented with metabarcoding samples obtained with other collecting methods such as Winkler, Malaise or light traps targeting alates^81^. Metabarcoding with Berlese-Tullgren may also provide additional data about non-target soil taxa not considered in this study. Sample processing can be simplified by obtaining bulk soil samples without extracting arthropods, although in this case the validity of analyses at the species level would need to be ascertained^20^. Overall, metabarcoding emerges as a method of choice for the long-term monitoring of the elusive biodiversity in tropical soils.

## Supplementary Information

Supplementary Information.

## Data Availability

The field protocol was deposited at protocols.io (https://doi.org/10.17504/protocols.io.bj9gkr3w). Sequences and BINs used as reference libraries are publicly accessible in BOLD projects as indicated in the text, and also deposited in GenBank databases under accession numbers KP845288-KP849461, KU745531-KU745532, KX072335-KX072563, MF922335-MF970719, MG030727, MK758129-MK770080, MN345316-MN621065 and MT357731. Details about sampling locations and a full list of species and their BINs are available in Ref.^33^. Raw sequences were deposited at the Sequence Read Archive (https://www.ncbi.nlm.nih.gov/bioproject/PRJNA668155).
