# Supplementary material for: Comparison of traditional and DNA metabarcoding samples for monitoring tropical soil arthropods (Formicidae, Collembola and Isoptera)
Source: Sci Rep. 2022 Jun 24;12:10762. doi: 10.1038/s41598-022-14915-2 (PMC9232565; doi:10.1038/s41598-022-14915-2)
Supplement: Supplementary file 1 — Supplementary Information. [file 41598_2022_14915_MOESM1_ESM.docx]

**Comparison of traditional and metabarcoding samples for monitoring tropical soil arthropods**

**(Formicidae, Collembola and Isoptera)**

Yves Basset, Mehrdad Hajibabaei, Michael Wright, Anakena M. Castillo, David A. Donoso, Simon T. Segar, Daniel Souto, Dina Soliman, Tomas Roslin, M. Alex Smith, Greg P.A. Lamarre, Luis F. De León, Thibaud Decaëns, José G. Palacios-Vargas, Gabriela Castaño-Meneses, Rudolf H. Scheffrahn, Marleny Rivera, Filonila Perez, Ricardo Bobadilla, Yacksecari Lopez, José Alejandro Ramirez, Maira Montejo Cruz, Angela Arango Galván

& Héctor Barrios

**Supplementary information**

**Appendix S1.** Supplementary methods.

**Appendix S2.** Supplementary methodological considerations.

**Table S1.** Analytical parameters used in the mBRAVE platform.

**Table S2.** Sensitivity analysis with mBrave parameters and the data of the present study.

**Table S3.** Distribution of post filter reads.

**Table S4.** Summary of run results.

**Table S5.** Numbers of reads in BINs, of unique BINs and reads in OTUs, detailed by arthropod orders and sampling location.

**Table S6.** Number of OTUs and complexes of focal species detected for three treatments.

**Table S7.** In silico PCR results using EcoPCR.

**Fig. S1.** Euler diagrams indicating the number of species with BINs detected by traditional and metabarcoding samples, and by both methods for ants, springtails and termites.

**Fig. S2.** Accumulation of species richness vs. the number of samples for common species of ants, springtails and termites.

**Fig. S3.** Plot of sample locations in the first two axes of the Procrustes rotation for all species of Formicidae, Collembola and Isoptera and for common species of the same taxa.

**Appendix S1. Supplementary methods.**

***Study site and field sampling***

Barro Colorado Island (BCI) receives an average annual rainfall of 2,645mm, with an annual average daily maximum and minimum air temperatures of 31.0°C and 23.6°C, respectively (<http://biogeodb.stri.si.edu/physical_monitoring/research/barrocolorado>). All paired samples used in this local study were 50-450m distant from each other and were dispersed over an area of ca 60ha on BCI. The two sampling events during the dry and wet season in 2017 accounted for insect seasonality in BCI soils (Levings & Windsor, 1982). After collection in the field, samples were stored in ice boxes and transported to the Smithsonian Tropical Research Institute. We restricted our sampling to the first 5cm of the litter/soil as tropical soils tend to be shallow, with most of the faunal diversity concentrated in the top few centimeters. For example, Ryder Wilkie et al. (2007) collected 93% of hypogeic ant species in Ecuador within the top 12.5 cm of rainforest soil.

### Processing of traditional samples

Specimens of ants and termites were deposited in the collections of the ForestGEO Arthropod Initiative, those of springtails in the collections of the Laboratorio de Ecología y Sistemática de Microartrópodos. A complete list of species and their BINs is provided in Basset et al. (2020).

***DNA metabarcoding***

Reactions had a standard mix of 17.5μL molecular grade water, 2.5μL 10x reaction buffer (200mM Tris HCl, 500mM KCl, pH 8.4), 1.0μL MgCl2 (50mM), 0.5μL dNTPs (10mM), 0.5μL forward primer (10mM), 0.5μL reverse primer (10mM), 0.5μL Platinum Taq DNA polymerase (5U/µL) (Life Technologies; Burlington, Ontario, Canada), and 2.0μL DNA template, for a total of 25μL per reaction. Two libraries were prepared per sample, one for each COI fragment and pooled after normalization. Sequencing was done over three partial MiSeq runs at approximately equal sequence coverage. Illumina adapters were removed by the sequencer before uploading sequence reads to mBRAVE.

***Bioinformatics***

*Paired end parameters*. Usually paired-end reads are merged as to get a better ‘average’ sequence and identify

errors. However, in our study, we were expecting very high matches to mBRAVE datasets with variation within a BIN

being small. Hence, the pooled option of mBRAVE makes sense where all target sequences are known and OTUs

are gathered into BOLD BINs. In other terms, pooling sequences allowed us to retain reads that would not be

kept through merging. Although this may result in using smaller or lower quality fragments, this is then mitigated

by subsequent quality control filtering. Additionally, since our interest was to detect taxa based on known BINs,

unpaired reads could still be matched with BINs from our mBRAVE datasets. Our sensitivity analysis, where we

varied most mBRAVE parameters one by one to optimize the greatest number of BINs recovered, confirmed that

the pooled option generated a superior number of BINs than the merged option (Table S2).

*Trimming parameters*. We trimmed all sequences from a fixed length of 25bp, rather than targeting

specific primer sequence with an additional tool like cutadapt (Martin, 2011). As our aim was to use the mBRAVE

platform with tailored BOLD datasets, cuadapt was less an option. The ‘Primer Masking’ function of mBRAVE is the

equivalent of cutadapt, but it was not implemented as of February 2022 (but will be in the future). However, the

incidence of primer length variation in Illumina Miseq data is incredibly low due to the low incidence of indels

(insertion–deletion mutations; Schirmer et al., 2015). Hence, our length-based trimming was appropriate. Note also

that the mBRAVE parameter “Trim reads to length specified” in Table S1 was set to 500bp meaning that any

read larger than 500bp would be trimmed. In the context of our analyses this would never be the case, as we

amplified fragments of maximum 310bp.

*Denoising.* Denoising parameters in mBRAVE correspond to the pre-clustering threshold, which was not used in

our analysis. However, denoising has limited impact when picking OTUs with a closed-reference approach (i.e.,

where sequences are identified prior to OTU generation), such as in our study. Closed-reference is superior to

de novo or open-reference, when a comprehensive reference library is available (McDonald et al. 2015).

*Tailored datasets*. The BOLD datasets that we used for analyses are currently the best one available for ants, springtails and termites from BCI. For ants we used data from the BOLD project BCIFO with 2,508 sequences including 413 BINs that represent 95% of ant species inhabiting the soils of BCI (D. Donoso et al., unpubl. data). The data were not so exhaustive for springtails but still reasonable. We used data from the BOLD project BCICL with 150 sequences and 53 BINs. For termites we used the BOLD project BCIIS with 234 sequences and 40 BINs. These three BOLD projects include only species collected on BCI and were grouped into the BOLD dataset DS-BCI1. We further combined the BOLD dataset TER including Neotropical termites with BOLD project BCIIS to create the dataset DS-TER2 with 1307 sequences and 408 BINs. In bioinformatic analyses we searched datasets for BINs and taxonomic assignment in the following order: (1) DS-BCI1, (2) DS-TER2, (3) DS-BCIARTH, (4) SYS-CRLINSECTA, (5) SYS-CRLNONINSECTARTH and (6) SYS-MBRAVEC (in total 584,329 BINs searched, details in Table S1). In the rare cases of multiple taxonomic identifications for the same BIN, we used names as available in the ForestGEO collections and databases.

***Statistical analyses***

*Question1: similarity between traditional* *and metabarcoding samples*

To help visualize the results of the rarefaction analyses, we extrapolated sampling curves of species richness with the package “iNEXT” (Hsieh et al., 2016) to about twice the number of samples (i.e., 200 instead of 95). We also computed an estimate of total species richness for each sample type and taxon with iNEXT. The non-metric multidimensional scaling (NMDS) was performed using Jaccard distance, as appropriate for presence-absence data.

*Question 3: seasonal shifts*

We fitted ordinary least squares regressions between the seasonal difference in traditional samples and that in metabarcoding samples. We assumed a linear difference between seasons, because a good linear relationship existed for the abundance of species in traditional samples collected during the wet and dry season (F_1,43_ = 40.9, p < 0.0001; data in Basset et al., 2020).

*Question 4: correlation between biomass and read frequency*

Body weight was estimated using the equation of Schoener (1980) for ants of the Guapiles rainforest in Costa Rica:

ln(weight)= ln(0.021) + 2.31*ln(body length)

**References**

Basset, Y. et al. Enemy-free space and the distribution of ants, springtails and termites in the soil of one tropical rainforest. *Eur. J. Soil Biol.* **99**, 103193 (2020).

Hsieh, T.C., Ma, K.H., & Chao, A. iNEXT: An R package for rarefaction and extrapolation of species diversity (Hill numbers). *Methods Ecol. Evol.* **7**, 1451–1456 (2016).

Levings, S.C., & Windsor, D.M. Seasonal and annual variation in litter arthropod populations. In E. G. Jr Leigh, A. S. Rand, & Windsor, D.M. (Eds.), *The Ecology of a Tropical Forest Seasonal Rhythms and Long-term Changes* (pp. 355–388). Washington D.C.: Smithsonian Institution Press (1982).

Martin, M. Cutadapt removes adapter sequences from high-throughput sequencing reads. *EMBnet* **17**, 10-12 (2011).

McDonald, D., Birmingham, A., & Knight, R. Context and the human microbiome. *Microbiome* **3**, 1-8 (2015).

Ryder Wilkie, K.T., Mertl, A.L., Traniello, J.F.A. Biodiversity below ground: Probing the subterranean ant fauna of Amazonia. *Naturwissenschaften* **94**, 725–731 (2007).

Schirmer, M. et al. Insight into biases and sequencing errors for amplicon sequencing with the Illumina MiSeq platform. *Nucleic Acids Res*. **43**, e37-e37 (2015).

Schoener, T. W. Length-weight regressions in tropical and temperate forest-understory insects. *Annals Ent. Soc. America* **73**, 106–109 (1980).

**Appendix S2.** **Supplementary methodological considerations.**

*Remaining OTUs not assigned to BINs*

OTUs not assigned to BINs and including only a few reads are more likely to be artefactual (e.g., Wang et al., 2019). Removing these problematic OTUs is challenging (Auer et al., 2017) and beyond the scope of this contribution, which is concerned about detecting known BINs, and not about estimating OTU richness. However, a common procedure includes applying an abundance-filter to the number of OTU sequences. For example, Elbrecht and Leese (2017) removed all OTUs with a minimum number of sequences corresponding to 0.003% of the total number of sequences. Other authors advised 0.005% of total sequence as the minimum threshold to exclude these problematic OTUs (Bokulich et al., 2013; Auer et al., 2017; Pauvert et al., 2019). We applied these two abundance-filters to our OTU data (Table S6). The tentative results suggest that while a few extra OTUs of Formicidae could be detected, these may eventually be related to a few BINs in five complexes of species, including that for *Tranopelta gilva*, which contains up to 91% of all OTUs retained at the 0.005% threshold (Table S6). As suggested by Table S6, the situation could be rather different for both Collembola and Isoptera, as more species could probably be detected if we had better DNA reference libraries. For Collembola, we lack an authoritative DNA reference library for BCI and for Isoptera, the taxonomy of the soldierless Apicotermitinae is rather challenging (please see next section). Further sequencing and taxonomic work targeting complexes of species such as those listed in Table S6 may therefore improve the detection of the focal taxa on BCI by metabarcoding.

*Limitations of the study*

Since our study explicitly relied on a comparison between traditional samples (treated as the “truth” against which DNA-based sample descriptors were compared), we should hurry to raise a few methodological limitations and caveats.

The first possible limitation in our study includes taxonomic challenges involved in sorting the diverse material collected. Out of the 426 species of Formicidae known of BCI, 39% can be considered as cryptic species (i.e., difficult to sort morphologically but with distinct BINs) and cannot currently be assigned to a proper binomial name (D. Donoso et al., unpubl. data). Arguably some of these species can be sorted morphologically with expert opinion but many taxonomic challenges remain. Sorting springtails is an expert task involving slide preparation and the study of chaetotaxy. Only 34 of the 104 species known on BCI have currently BINs and 29% of the species with BINs have multiple ones (Basset et al., 2020). For termites, taxonomic challenges concern mostly the soldierless Apicotermitinae, which represent 22.6% of the 62 species known on BCI (Y. Basset et al., unpubl. data). All soldierless species are best identified using characters of the enteric valve and molecular data. Because of all these challenges and the fact that not all specimens collected were systematically sequenced, we can expect biases in our traditional samples. If many BINs are missed in sorting traditional samples but are picked up by metabarcoding, then serious mismatch can occur between the two types of samples. This was not the case in this study as the number of probable false positive was rather low. Further, these cases can be flagged in results with a good knowledge of the local fauna. The case of the “*Ectatomma ruidium*” complex is a good example (Table 3; Meza-Lázaro et al., 2018). Note that sampling effort (100 samples) was adequate to survey at least the most common species present locally.

Second, we assumed that traditional samples represent true abundance and faunal composition, and that paired metabarcoding samples represent a surrogate of these variables. The similarity between traditional and metabarcoding samples is best studied by using “mock communities” (Gibson et al.*,* 2014; Braukmann et al., 2019), but assembling mock communities with cryptic species for the three taxa studied, which are among the dominant groups in the soil, would be rather challenging (but see Saitoh et al., 2016 for springtails). Further, the focus on particular species and the low species richness in these mock communities has little comparison with the rich diversity of tropical samples (Creedy et al., 2019). As for our paired samples, we can expect discrepancies between samples obtained only 10cm apart because soil organisms, such as ants, can be very patchy when studied at the 1m^2^ scale (Kaspari, 1996). To reduce this effect, whenever possible, our analyses emphasized data in which samples were grouped per location, as to evaluate differences at a higher scale. We also considered a stringent rule to define probable false positive/negative species for this reason. In other terms, if a species was reasonably common (the target of our monitoring scheme) at a certain location (5 samples pooled) and identified in traditional samples, then it should also be detected among the metabarcoding samples (5 samples pooled) originating from that same location. However, we cannot fully discount spatial differences in faunal composition between traditional and metabarcoding samples.

Third, our study concerns three of the dominant soil taxa but did not include other important groups such as earthworms, Acari and nematodes. We showed that different patterns existed among our focal taxa, particularly regarding the efficiency of metabarcoding. Hence, we expect that taxa not covered by this study may exhibit different patterns than those presented here.

Fourth, a possible complication is that ant workers, which are rather mobile, prey on springtails and termites, although these interactions are not very specialized (Basset et al., 2020; Tuma et al., 2020). In some cases, ant workers may have ingested prey outside the collecting area, but prey DNA may be detected in metabarcoding samples, generating false positive records when comparing traditional samples (Oliverio et al., 2018). How common this situation may be is difficult to assess for the present study (but see Fayle et al., 2015). Another way of generating false positive and discrepancies between traditional and metabarcoding information is the prevalence of juveniles in samples, which typically are difficult or impossible to identify in traditional samples but can be detected in metabarcoding samples. This is more likely to happen for springtails, as juveniles of social insects are always present with adults.

As a final, long-term challenge, the precise methodology of metabarcoding remains in flux (Creedy et al.*,* 2019). New advances in sequencing technology and bioinformatics are enhancing the applicability of this approach in various ecological settings. For example, ultra-deep sequencing can aid in resolving biodiversity composition and overcoming some of the biases in taxonomic coverage (Singer et al., 2019). With regard to the long-term monitoring of a diverse and poorly known group such as arthropods with metabarcoding, one may fear that once a pipeline protocol has been well established and reasonable time-series obtained, that future improvements in technology, such as switch to metagenomic sequencing (Porter & Hajibabaei, 2018), more efficient DNA extraction protocols and/or PCR, more efficient primers, may yield rather different results and time-series. This is not a challenge for species previously undetected, but rather for species detected in previous time-series that may be detected with a higher or lower detection probability in newer laboratory protocols. Fortunately, several methods exist to account for changes in methodology and may ensure the comparability of data, including data weighting, standardization, year-specific deviations from the scheme average, estimates of population growth rate per year or parallel monitoring for a reduced time to align time-series (Buckland et al., 2005; Henry et al., 2008; Magurran et al., 2010).

**References**

Auer, L. et al. Analysis of large 16S rRNA Illumina data sets: Impact of singleton read filtering on microbial community description. *Mol. Ecol. Res.* **17**, e122-e132 (2017).

Basset, Y. et al. Enemy-free space and the distribution of ants, springtails and termites in the soil of one tropical rainforest. *Eur. J. Soil Biol.* **99**, 103193 (2020).

Bokulich, N.A. et al. Quality-filtering vastly improves diversity estimates from Illumina amplicon sequencing. *Nature Methods* **10**, 57–59 (2013).

Braukmann, T.W. et al. Metabarcoding a diverse arthropod mock community. *Mol. Ecol. Res*. **19**, 711–727 (2019).

Buckland, S.T. et al. Monitoring change in biodiversity through composite indices. *Phil. Trans. Royal Soc. B: Biol. Sci.* **360**, 243–254 (2005).

Creedy, T. J., Ng, W. S., & Vogler, A. P. Toward accurate species‐level metabarcoding of arthropod communities from the tropical forest canopy. *Ecol. Evol.* **9**, 3105–3116 (2019).

Elbrecht, V., & Leese, F. Validation and development of COI metabarcoding primers for freshwater macroinvertebrate bioassessment. *Front. Envir. Science* **5**, 11 (2017).

Fayle, T. M. et al. Detection of mitochondrial COII DNA sequences in ant guts as a method for assessing termite predation by ants. *PLoS One* **10**, e0122533 (2015).

Gibson, J. et al. Simultaneous assessment of the macrobiome and microbiome in a bulk sample of tropical arthropods through DNA metasystematics. *PNAS* **111**, 8007–8012 (2014).

Henry, P. Y. et al. Integrating ongoing biodiversity monitoring: potential benefits and methods. *Biodiv. Conserv.* **17**, 3357–3382 (2008).

Kaspari, M. Litter ant patchiness at the 1-m^2^ scale: disturbance dynamics in three Neotropical forests. *Oecologia* **107**, 265–273 (1996).

Magurran, A.E. et al. Long-term datasets in biodiversity research and monitoring: assessing change in ecological communities through time. *TREE* **25**, 574—582 (2010).

Meza-Lázaro, R.N. et al. Extensive mitochondrial heteroplasmy in the neotropical ants of the *Ectatomma ruidum* complex (Formicidae: Ectatomminae). *Mitochondrial DNA Part A* **29**, 1203–1214 (2018).

Oliverio, A.M. et al. A DNA metabarcoding approach to characterize soil arthropod communities. *Soil Biol. Biochem.* **125**, 37–43 (2018).

Pauvert, C. et al. Bioinformatics matters: The accuracy of plant and soil fungal community data is highly dependent on the metabarcoding pipeline. *Fungal Ecol.* **41**, 23–33 (2019).

Porter, T.M., & Hajibabaei, M. Scaling up: A guide to high‐throughput genomic approaches for biodiversity analysis. *Mol. Ecol.* **27**, 313–338 (2018).

Saitoh, S. et al. A quantitative protocol for DNA metabarcoding of springtails (Collembola). *Genome* **59**, 705–723 (2016).

Singer, G.A.C. et al. Comprehensive biodiversity analysis via ultra-deep patterned flow cell technology: a case study of eDNA metabarcoding seawater. *Sci. Rep.* **9**, 1–12 (2019).

Tuma, J., Eggleton, P., & Fayle, T.M. Ant‐termite interactions: an important but under‐explored ecological linkage. *Biol. Rev.* **95**, 555–572 (2020).

Wang, X. et al. The biodiversity benefit of native forests and mixed‐species plantations over monoculture plantations. *Diver. Distrib.* **25**, 1721–1735 (2019).

**TABLES**

**Table S1.** Analytical parameters used in the mBRAVE platform. Tasks as defined in Creedy et al. (2021).

| **Parameters** | **Description** | **Value** | **Tasks** | **Tool** | **Examples of alternative pipeline tools** |
| --- | --- | --- | --- | --- | --- |
| **1. Paired End Parameters** ^1^ |  |  | Read preparation:  pair merging/pooling | PEAR: Zhang et al. 2013 | join_paired_ends with QIIME: Caporaso et al. 2010; pimba_prepare with PIMBA: Oliveira et al. 2021 |
| Paired End Merge Uploads | Pooled: pools paired-end reads. Merged: assembles overlapping paired-end reads ^2^ | Pooled |  |  |  |
| Assembler Min Overlap (Merged) | Minimum overlap needed for each pair of reads | (20bp) |  |  |  |
| Assembler Max Substitutions (Merged) | Maximum substitutions allowed in the overlap for each pair of reads | (5bp) |  |  |  |
| **2. Trimming parameters** ^1^ |  |  | Read preparation: trimming  of primers and filtering (length filtering) | Custom mBRAVE code | -m LENGTH with cutadapt: Martin 2011; split_libraries with QIIME: Caporaso et al. 2010; pimba_prepare with PIMBA: Oliveira et al. 2021 |
| Trim Front | Read length to be trimmed from front, useful for removing primers | 25bp |  |  |  |
| Trim End | Read length to be trimmed from end, useful for removing primers | 25bp |  |  |  |
| Trim Length | Trim reads to length specified | 500bp |  |  |  |
| Primer Masking | Beta version, not implemented as of February 2022 | Off |  |  |  |
| **3. Filtering Parameters** |  |  | Read Preparation:  quality trimming | Custom mBRAVE code based on  quality values | -q with cutadapt: Martin 2011; split_libraries with QIIME: Caporaso et al. 2010; pimba_prepare with PIMBA: Oliveira et al. 2021 |
| Min QV (quality value) | Filter out reads below the Minimum QV | 20qv |  |  |  |
| Min Length | Filter out reads below the Minimum Length | 100bp |  |  |  |
| Max Bases with Low QV (<20) | Filter out reads with low QV by set threshold | 25% |  |  |  |
| Max Bases with Ultra Low QV (<10) | Filter out reads with ultra low QV by set threshold | 1% |  |  |  |
| **4. Other Parameters** |  |  | Filtering: denoising (pre-clustering), chimera & frequency filtering | Combination of: VSEARCH: Rognes et al. 2016 and EMBOSS (Needleman-Wunsch algorithm): Madeira et al. 2019 | cluster_otus with USEARCH: Edgar 2010; uchime with VSEARCH: Rognes et al. 2016; identify_chimeric_seqs with QIIME: Caporaso et al. 2010; pimba_run with PIMBA: Oliveira et al. 2021 |
| Pre-Clustering Threshold ^1^ | Cluster reads by similarity | None ^1^ |  |  |  |
| ID Distance Threshold | Threshold for ID Engine match maximum distance | 3% |  |  |  |
| Exclude From OTU Threshold | Threshold for excluding reads from OTU generation (spurious haplotypes) ^3^ | 3% |  |  |  |
| Minimum OTU Size ^4^ | Minimum amount of reads required to create an OTU | 1 |  |  |  |
| OTU Threshold | Threshold for maximum distance inside a generated OUT | 2% |  |  |  |
| **5. BOLD datasets** ^1^ |  |  | Data Generation: referenced taxonomic assignment | Custom BOLD databases tailored for this project (Ratnasingham & Hebert 2007) | assign_taxonomy with QIIME Caporaso et al. 2010; pimba_run with PIMBA (specify taxonomy file): Oliveira et al. 2021 |
| (1) DS-BCI1 | BOLD projects BCIFO, BCICL and BCIIS, 506 BINs Scale: Barro Colorado Island | - |  |  |  |
| (2) DS-TER2 | Neotropical termites, 408 BINs. Scale: Neotropical | - |  |  |  |
| (3) DS-BCIARTH | Focal groups studied by the ForestGEO Arthropod Initiative on BCI, 2,571 BINs  Scale: Barro Colorado Island | - |  |  |  |
| (4) SYS-CRLINSECTA | System reference library for insects, 516,841 BINs  Scale: worldwide | - |  |  |  |
| (5) SYS-CRLNONINSECTARTH | System reference library for arthropods excluding insects, 63,823 BINs. Scale: worldwide | - |  |  |  |
| (6) SYS-MBRAVEC | System reference library for contaminants based on reagent production, 75 BINs. Scale: worldwide | - |  |  |  |

^1^ See Appendix S1

^2^ The merged paired-end reads option is for overlapping paired-end reads, while the pooled option will pool paired-end reads, which are treated as one fastq file without merging. See Appendix S1.

^3^ Excluding reads from OTU generation when sequencing error introduces spurious haplotypes (chimeras and/or sequence errors).

^4^ For more details see Appendix S1 and Table S5.

**References:**

Caporaso, J.G. et al. QIIME allows analysis of high-throughput community sequencing data. *Nature Methods* **7**, 335-336 (2010).

Creedy, T.J. et al. Coming of age for COI metabarcoding of whole organism community DNA: Towards bioinformatic harmonisation. *Mol. Ecol. Res*. doi:10.1111/1755-0998.13502 (2021).

Edgar, R.C. Search and clustering orders of magnitude faster than BLAST. *Bioinformatics* **26**, 2460-2461 (2010).

Madeira, F. et al. The EMBL-EBI search and sequence analysis tools APIs in 2019. *Nucleic Acids Res*. **47**, W636-W641 (2019).

Martin, M. Cutadapt removes adapter sequences from high-throughput sequencing reads. *EMBnet* **17**, 10-12 (2011).

Oliveira, R.R. et al. Pimba: a pipeline for metabarcoding analysis. In *Brazilian Symposium on Bioinformatics* (eds. Stadler P.F et al.), pp. 106-116. Springer, Cham (2021).

Ratnasingham, S., & Hebert, P. BOLD: The Barcode of Life Data System (http://www. barcodinglife. org). *Mol. Ecol. Notes* **7**, 355-364 (2007).

Rognes, T. et al. VSEARCH: a versatile open source tool for metagenomics. *PeerJ* **4**, e2584 (2016).

Zhang, J. et al. PEAR: a fast and accurate Illumina Paired-End reAd mergeR. *Bioinformatics* **30**, 614-620 (2013).

**Table S2.** Sensitivity analysis with mBrave parameters and the data of the present study. Parameters varying from mBrave defaults are indicated (greyed cells) for each of 11 main analyses performed. In the context of our study, optimal results should include a high number of credible (i.e., no false positives) BINs detected, as well as high number of records detected of the three focal taxa. mBrave parameters as detailed in Table S1.

| **mBRave parameter** | **Default analysis** | **Optimal analysis** | **Analysis**  **1** | **Analysis**  **2** | **Analysis**  **3** | **Analysis 4** | **Analysis**  **5** | **Analysis**  **6** | **Analysis 7** | **Analysis 8** | **Analysis**  **9** | **Analysis**  **10** |  |
| --- | --- | --- | --- | --- | --- | --- | --- | --- | --- | --- | --- | --- | --- |
| (Short description of analysis) | Defaults mBrave | Optimal | No trim of primers | Min QV=20 | Min length 200bp | Max Low QV 25% | ID OTU threshold 2% | ID OTU threshold 4% | Min OTU 10 | Min OTU 100 | Paired End merged | Trim front/end: 25/0 |  |
| Paired End Merge Uploads | Pooled | Pooled | Pooled | Pooled | Pooled | Pooled | Pooled | Pooled | Pooled | Pooled | Merged | Pooled |  |
| Assembler Min Overlap | 20bp | (20bp) | (20bp) | (20bp) | (20bp) | (20bp) | (20bp) | (20bp) | (20bp) | (20bp) | 20bp | (20bp) |  |
| Assembler Max Substitutions | 5bp | (5bp) | (5bp) | (5bp) | (5bp) | (5bp) | (5bp) | (5bp) | (5bp) | (5bp) | 5bp | (5bp) |  |
| Trim Front | 50bp | 25bp | 0bp | 50bp | 50bp | 50bp | 50bp | 50bp | 50bp | 50bp | 50bp | 25bp |  |
| Trim End | 0bp | 25bp | 0bp | 0bp | 0bp | 0bp | 0bp | 0bp | 0bp | 0bp | 0bp | 0bp |  |
| Trim Length | 500bp | 500bp | 500bp | 500bp | 500bp | 500bp | 500bp | 500bp | 500bp | 500bp | 500bp | 500bp |  |
| Primer Masking | Off | Off | Off | Off | Off | Off | Off | Off | Off | Off | Off | Off |  |
| Min QV (quality value) | 10qv | 20qv | 10qv | 20qv | 10qv | 10qv | 10qv | 10qv | 10qv | 10qv | 10qv | 10qv |  |
| Min Length | 100bp | 100bp | 100bp | 100bp | 200bp | 100bp | 100bp | 100bp | 100bp | 100bp | 100bp | 100bp |  |
| Max bases with low QV (<20) | 4% | 25% | 4% | 4% | 4% | 25% | 4% | 4% | 4% | 4% | 4% | 4% |  |
| Max bases with  ultra low QV(<10) | 1% | 1% | 1% | 1% | 1% | 1% | 1% | 1% | 1% | 1% | 1% | 1% |  |
| Pre-Clustering Threshold | None | None | None | None | None | None | None | None | None | None | None | None |  |
| ID Distance Threshold | 3% | 3% | 3% | 3% | 3% | 3% | 2% | 4% | 3% | 3% | 3% | 3% |  |
| Exclude From OTU Threshold | 3% | 3% | 3% | 3% | 3% | 3% | 2% | 4% | 3% | 3% | 3% | 3% |  |
| Minimum OTU Size | 1 | 1 | 1 | 1 | 1 | 1 | 1 | 1 | 10 | 100 | 1 | 1 |  |
| OTU Threshold | 2% | 2% | 2% | 2% | 2% | 2% | 2% | 2% | 2% | 2% | 2% | 2% |  |
| **Results** |  |  |  |  |  |  |  |  |  |  |  |  |  |
| BINs detected (n) | 364 | 396 | 270 | 364 | 342 | 395 | 333 | 419 | 364 | 364 | 336 | 311 |  |
| Sequences (n) | 5758139 | 6188608 | 4191489 | 5758139 | 5757936 | 6188613 | 5538122 | 6183615 | 5758139 | 5758139 | 3513737 | 4215657 |  |
| Preclusters (n) | 1037475 | 1278135 | 1257752 | 1037475 | 1037296 | 1278140 | 953680 | 1123352 | 1037475 | 1037475 | 515732 | 932996 |  |
| Mean sequence length (bp) | 233.82 | 233.00 | 285.20 | 233.82 | 240.8 | 233.38 | 233.52 | 235.11 | 233.82 | 233.82 | 255.46 | 257.14 |  |
| Arthropod orders (n) | 29 | 29 | 25 | 29 | 26 | 29 | 29 | 28 | 29 | 29 | 26 | 26 |  |
| Arthropod families (n) | 97 | 104 | 79 | 97 | 86 | 103 | 94 | 100 | 97 | 97 | 89 | 87 |  |
| Total records with BINs (n) | 1756 | 2021 | 1388 | 1757 | 109 | 2021 | 1668 | 1879 | 1756 | 1756 | 1444 | 1477 |  |
| Ant records with BINs (n) | 276 | 322 | 241 | 276 | 12 | 321 | 276 | 281 | 276 | 276 | 243 | 256 |  |
| Collembola records with BINs (n) | 324 | 364 | 306 | 324 | 9 | 364 | 320 | 307 | 324 | 324 | 265 | 287 |  |
| Termite records with BINs (n) | 301 | 343 | 246 | 300 | 37 | 344 | 284 | 343 | 301 | 300 | 211 | 250 |  |
| **Quality of analysis** | Good | Best | Poor | Good | Poor | Near best | Poor | Not retained | Good | Good | Poor | Poor |  |
| **Notes** | Defaults mBrave | Optimal | Information loss | Similar to defaults | Information loss | Better than defaults | Information loss | (1) | Similar to defaults | Similar to defaults | Information loss | Information loss |  |

(1) BINs gained for termites over the optimal analysis are highly dubious (false positives). Records of focal groups are also fairly low.

**Table S3.** Distribution of the post filter reads in OTUs and BINs, after filtering a total 30.46 million reads obtained from 95 soil samples at BCI. Of the total number of reads, 0.13 million were chimeras.

| **Variable** | **Total reads**  **(million)** | **No. reads in OTUs (million)** | **No. read in BINs (million)** |
| --- | --- | --- | --- |
| Post filter reads | 15.96 | 9.77 | 6.19 |
| Post filter reads - arthropods | 9.56 | 3.40 | 6.16 |
| Post filter reads - focal taxa^1^ | 3.95 | 0.48 | 3.47 |

^1^Ants, springtails and termites

**Table S4.** Summary of run results.

| **Run name** | **SampleCode (Location-Season-Distance)** | **Reads uploaded (n)** | **Post filter reads (n)** | **Mean length (bp)** | **Mean QV** | **Mean GC %** | **Dereplicated reads (n)** | **Preclustered reads (n)** | **Reads in BINs (n)** | **Reads in OTUs (n)** | **Reads in chimera (n)** | **BINs Arthropoda (n)** | **BINs Formicidae (n)** | **BINs Collembola (n)** | **BINs Isoptera (n)** | **Total OTUs (n)** | **OTUs Arthropoda (n)^1^** | **OTUs Formicidae (n)^1^** | **OTUs Collembola (n)^1^** | **OTUs Isoptera (n)^1^** |
| --- | --- | --- | --- | --- | --- | --- | --- | --- | --- | --- | --- | --- | --- | --- | --- | --- | --- | --- | --- | --- |
| Basset-Panama-BR5-S-B-ARM1-050-MAR17_S47 | ARM1-MAR2017-350 | 135830 | 34987 | 250.6 | 36.3 | 46.6 | 7548 | 7548 | 0 | 34891 | 96 | 0 | 0 | 0 | 0 | 116 | 0 | 0 | 0 | 0 |
| Basset-Panama-BR5-S-B-ARM1-350-MAR17_S49 | ARM1-MAR2017-350 | 218866 | 69419 | 238.8 | 36.4 | 48.8 | 21340 | 21340 | 2761 | 65643 | 1015 | 5 | 0 | 1 | 1 | 528 | 10 | 0 | 3 | 3 |
| Basset-Panama-BR5-S-B-ARM1-500-MAR17_S50 | ARM1-MAR2017-500 | 243342 | 113771 | 245.5 | 36.4 | 42.6 | 37906 | 37906 | 1770 | 107675 | 4326 | 10 | 1 | 1 | 3 | 1192 | 32 | 4 | 5 | 0 |
| Basset-Panama-BR5-S-B-ARM2-050-MAR17_S51 | ARM2-MAR2017-050 | 227346 | 109588 | 251.0 | 36.4 | 44.9 | 21601 | 21601 | 16 | 109510 | 62 | 9 | 1 | 0 | 4 | 336 | 0 | 0 | 0 | 0 |
| Basset-Panama-BR5-S-B-ARM2-200-MAR17_S52 | ARM2-MAR2017-200 | 79689 | 13439 | 250.9 | 35.7 | 43.9 | 4120 | 4120 | 21 | 13417 | 1 | 4 | 0 | 0 | 1 | 80 | 1 | 0 | 0 | 0 |
| Basset-Panama-BR5-S-B-ARM2-250-MAR17_S53 | ARM2-MAR2017-250 | 271086 | 92718 | 250.7 | 36.1 | 43.5 | 20984 | 20984 | 3 | 91576 | 1139 | 2 | 0 | 0 | 0 | 243 | 1 | 0 | 0 | 0 |
| Basset-Panama-BR5-S-B-ARM2-350-MAR17_S54 | ARM2-MAR2017-350 | 203654 | 83621 | 224.3 | 36.2 | 41.6 | 24812 | 24812 | 45942 | 36766 | 913 | 3 | 0 | 0 | 2 | 1856 | 175 | 0 | 0 | 173 |
| Basset-Panama-BR5-S-B-ARM2-450-MAR17_S55 | ARM2-MAR2017-450 | 235774 | 108411 | 250.9 | 36.3 | 40.5 | 25072 | 25072 | 72152 | 35950 | 309 | 5 | 1 | 0 | 2 | 278 | 126 | 0 | 0 | 2 |
| Basset-Panama-BR5-S-B-ARM3-050-MAR17_S56 | ARM3-MAR2017-050 | 195706 | 42129 | 248.9 | 36.4 | 52.0 | 10981 | 10981 | 638 | 41077 | 414 | 4 | 0 | 0 | 3 | 196 | 9 | 0 | 0 | 1 |
| Basset-Panama-BR5-S-B-ARM3-200-MAR17_S57 | ARM3-MAR2017-200 | 178998 | 34815 | 245.7 | 36.5 | 56.2 | 7828 | 7828 | 5 | 34650 | 160 | 5 | 0 | 0 | 3 | 126 | 1 | 0 | 0 | 0 |
| Basset-Panama-BR5-S-B-ARM3-350-MAR17_S58 | ARM3-MAR2017-350 | 261032 | 147734 | 250.7 | 36.4 | 37.7 | 35532 | 35532 | 36448 | 106763 | 4523 | 6 | 0 | 3 | 1 | 922 | 409 | 0 | 2 | 1 |
| Basset-Panama-BR5-S-B-ARM3-400-MAR17_S59 | ARM3-MAR2017-400 | 257786 | 118433 | 250.9 | 36.3 | 42.8 | 30830 | 30830 | 66428 | 49024 | 2981 | 5 | 0 | 0 | 3 | 388 | 109 | 0 | 1 | 107 |
| Basset-Panama-BR5-S-B-ARM3-500-MAR17_S60 | ARM3-MAR2017-500 | 164604 | 45691 | 250.7 | 36.4 | 46.0 | 11765 | 11765 | 6403 | 39113 | 175 | 8 | 2 | 1 | 1 | 116 | 15 | 12 | 2 | 0 |
| Basset-Panama-BR5-S-B-ARM4-150-MAR17_S61 | ARM4-MAR2017-150 | 202320 | 68631 | 219.8 | 36.4 | 51.3 | 28385 | 28385 | 2749 | 64758 | 1124 | 7 | 1 | 2 | 1 | 4689 | 28 | 1 | 13 | 0 |
| Basset-Panama-BR5-S-B-ARM4-400-MAR17_S63 | ARM4-MAR2017-400 | 252426 | 101884 | 251.0 | 36.3 | 46.8 | 17708 | 17708 | 65830 | 35868 | 186 | 5 | 1 | 0 | 2 | 98 | 18 | 0 | 0 | 0 |
| Basset-Panama-BR5-S-B-ARM4-500-MAR17_S65 | ARM4-MAR2017-500 | 247662 | 62758 | 249.0 | 36.3 | 55.4 | 13635 | 13635 | 16 | 62573 | 169 | 5 | 1 | 0 | 1 | 165 | 0 | 0 | 0 | 0 |
| Basset-Panama-BR5-S-B-BAL1-050-MAR17_S66 | BAL1-MAR2017-050 | 245458 | 147143 | 250.9 | 36.4 | 38.6 | 21373 | 21373 | 12 | 147108 | 23 | 6 | 1 | 0 | 2 | 417 | 2 | 0 | 0 | 1 |
| Basset-Panama-BR5-S-B-BAL1-150-MAR17_S67 | BAL1-MAR2017-150 | 227652 | 62172 | 249.8 | 35.5 | 31.4 | 17467 | 17467 | 23 | 62114 | 35 | 6 | 1 | 0 | 2 | 531 | 243 | 0 | 0 | 1 |
| Basset-Panama-BR5-S-B-BAL1-350-MAR17_S68 | BAL1-MAR2017-350 | 167626 | 56427 | 250.8 | 36.3 | 42.8 | 11829 | 11829 | 2 | 56329 | 96 | 1 | 0 | 0 | 0 | 160 | 0 | 0 | 0 | 0 |
| Basset-Panama-BR5-S-B-BAL1-400-MAR17_S69 | BAL1-MAR2017-400 | 279370 | 162487 | 251.0 | 36.5 | 39.0 | 22062 | 22062 | 39619 | 122682 | 186 | 3 | 1 | 0 | 1 | 311 | 62 | 61 | 0 | 0 |
| Basset-Panama-BR5-S-B-DRA1-100-MAR17_S70 | DRA1-MAR2017-100 | 265140 | 132092 | 250.8 | 36.3 | 42.3 | 28477 | 28477 | 1E+05 | 12031 | 41 | 3 | 0 | 0 | 1 | 179 | 70 | 0 | 0 | 70 |
| Basset-Panama-BR5-S-B-DRA1-150-MAR17_S71 | DRA1-MAR2017-150 | 220142 | 57464 | 250.2 | 36.2 | 50.0 | 9041 | 9041 | 5 | 57455 | 4 | 2 | 1 | 0 | 1 | 128 | 0 | 0 | 0 | 0 |
| Basset-Panama-BR5-S-B-ARM4-200-MAR17_S62 | ARM4-MAR2017-200 | 280864 | 111423 | 250.5 | 36.3 | 43.4 | 25857 | 25857 | 9 | 110202 | 1212 | 3 | 1 | 0 | 1 | 320 | 0 | 0 | 0 | 0 |
| Basset-Panama-BR5-S-B-ARM4-450-MAR17_S64 | ARM4-MAR2017-450 | 240524 | 97365 | 249.8 | 36.3 | 43.1 | 23923 | 23923 | 11447 | 85186 | 732 | 8 | 1 | 0 | 3 | 432 | 98 | 0 | 0 | 4 |
| Basset-Panama-BR5-S-B-DRA1-350-MAR17_S72 | DRA1-MAR2017-350 | 229232 | 85517 | 251.0 | 36.2 | 36.7 | 14639 | 14639 | 5 | 85508 | 4 | 3 | 1 | 0 | 1 | 225 | 6 | 0 | 0 | 0 |
| Basset-Panama-BR5-S-B-DRA1-450-MAR17_S73 | DRA1-MAR2017-450 | 19498 | 94 | 244.9 | 35.8 | 47.3 | 79 | 79 | 8 | 86 | 0 | 5 | 0 | 1 | 1 | 35 | 2 | 0 | 0 | 0 |
| Basset-Panama-BR5-S-B-DRA1-500-MAR17_S74 | DRA1-MAR2017-500 | 33086 | 1673 | 250.8 | 36.2 | 64.2 | 523 | 523 | 7 | 1664 | 2 | 3 | 0 | 0 | 1 | 40 | 3 | 0 | 0 | 0 |
| Basset-Panama-BR5-S-B-WHE1-050-MAR17_S75 | WHE1-MAR2017-050 | 83668 | 23612 | 249.5 | 36.5 | 41.4 | 6270 | 6270 | 14994 | 8591 | 27 | 5 | 1 | 0 | 2 | 121 | 76 | 0 | 0 | 0 |
| Basset-Panama-BR5-S-B-WHE1-200-MAR17_S76 | WHE1-MAR2017-200 | 75784 | 8608 | 251.0 | 35.5 | 41.0 | 3505 | 3505 | 1247 | 7320 | 41 | 3 | 0 | 0 | 2 | 80 | 21 | 0 | 0 | 0 |
| Basset-Panama-BR5-S-B-WHE1-250-MAR17_S77 | WHE1-MAR2017-250 | 236376 | 84203 | 250.5 | 36.3 | 40.9 | 18559 | 18559 | 7 | 84183 | 13 | 4 | 0 | 0 | 1 | 261 | 1 | 0 | 0 | 0 |
| Basset-Panama-BR5-S-B-WHE2-450-MAR17_S78 | WHE2-MAR2017-450 | 127012 | 42322 | 251.0 | 36.2 | 41.4 | 10284 | 10284 | 20 | 42079 | 223 | 6 | 0 | 0 | 3 | 186 | 2 | 0 | 0 | 0 |
| Basset-Panama-BR5-S-B-WHE2-500-MAR17_S79 | WHE2-MAR2017-500 | 224272 | 107995 | 248.8 | 36.3 | 42.4 | 25374 | 25374 | 96523 | 10439 | 1033 | 11 | 1 | 0 | 4 | 565 | 141 | 0 | 0 | 1 |
| Basset-Panama-BR5-S-B-ZET1-050-MAR17_S80 | ZET1-MAR2017-050 | 115682 | 22483 | 250.6 | 36.4 | 58.4 | 5556 | 5556 | 5 | 22332 | 146 | 2 | 0 | 0 | 2 | 51 | 1 | 0 | 0 | 0 |
| Basset-Panama-BR5-S-B-ZET1-100-MAR17_S81 | ZET1-MAR2017-100 | 233730 | 109203 | 250.8 | 36.5 | 40.2 | 22350 | 22350 | 3 | 109007 | 193 | 3 | 1 | 0 | 0 | 274 | 1 | 0 | 0 | 0 |
| Basset-Panama-BR5-S-B-ZET1-250-MAR17_S82 | ZET1-MAR2017-250 | 224992 | 83265 | 250.9 | 36.3 | 49.8 | 14654 | 14654 | 52 | 83185 | 28 | 4 | 1 | 0 | 2 | 138 | 8 | 0 | 0 | 1 |
| Basset-Panama-BR5-S-B-ZET1-350-MAR17_S83 | ZET1-MAR2017-350 | 240784 | 141232 | 250.8 | 36.4 | 38.8 | 30857 | 30857 | 26 | 139084 | 2122 | 6 | 0 | 0 | 3 | 732 | 4 | 0 | 0 | 3 |
| Basset-Panama-BR5-S-B-ZET1-500-MAR17_S84 | ZET1-MAR2017-500 | 285096 | 172649 | 251.0 | 36.5 | 33.6 | 32398 | 32398 | 12 | 172366 | 271 | 3 | 1 | 0 | 1 | 914 | 229 | 0 | 0 | 0 |
| Basset-Panama-BR5-S-B-ARM1-200-MAR17_S48 | ARM1-MAR2017-200 | 198580 | 80492 | 251.0 | 36.4 | 34.3 | 15932 | 15932 | 0 | 80474 | 18 | 0 | 0 | 0 | 0 | 469 | 0 | 0 | 0 | 0 |
| Basset-Panama-BR5-S-B-ZET2-050-MAR17_S85 | ZET2-MAR2017-050 | 235924 | 100377 | 250.2 | 36.0 | 37.8 | 23231 | 23231 | 4 | 99292 | 1081 | 2 | 1 | 0 | 0 | 571 | 123 | 0 | 0 | 0 |
| Basset-Panama-BR5-S-B-ZET2-250-MAR17_S86 | ZET2-MAR2017-250 | 269204 | 140467 | 250.9 | 36.3 | 37.1 | 26461 | 26461 | 3 | 140415 | 49 | 1 | 1 | 0 | 0 | 863 | 1 | 0 | 0 | 0 |
| Basset-Panama-BR5-S-B-ZET2-300-MAR17_S87 | ZET2-MAR2017-300 | 186982 | 74764 | 250.9 | 36.4 | 34.0 | 17556 | 17556 | 41610 | 33061 | 93 | 7 | 2 | 2 | 1 | 234 | 88 | 84 | 3 | 0 |
| Basset-Panama-BR5-S-B-ZET2-500-MAR17_S88 | ZET2-MAR2017-500 | 47722 | 13708 | 251.0 | 36.3 | 42.7 | 2773 | 2773 | 2 | 13706 | 0 | 1 | 0 | 0 | 0 | 54 | 1 | 0 | 0 | 0 |
| Basset-Panama-CO1-S-ARM1-050-Dec2017_S1 | ARM1-DEC2017-050 | 312490 | 198041 | 239.6 | 36.7 | 37.8 | 47532 | 47532 | 98572 | 98890 | 579 | 27 | 8 | 4 | 3 | 2664 | 1205 | 63 | 6 | 28 |
| Basset-Panama-CO1-S-ARM1-250-Dec2017_S2 | ARM1-DEC2017-250 | 470354 | 351281 | 239.5 | 36.7 | 38.1 | 77741 | 77741 | 3E+05 | 81643 | 954 | 39 | 7 | 8 | 9 | 5782 | 1935 | 67 | 6 | 614 |
| Basset-Panama-CO1-S-ARM1-300-Dec2017_S3 | ARM1-DEC2017-300 | 108971 | 28068 | 231.6 | 36.8 | 30.2 | 6402 | 6402 | 26794 | 1259 | 15 | 28 | 6 | 4 | 4 | 629 | 360 | 295 | 3 | 6 |
| Basset-Panama-CO1-S-ARM1-450-Dec2017_S4 | ARM1-DEC2017-450 | 406697 | 298932 | 233.7 | 36.8 | 32.7 | 74183 | 74183 | 2E+05 | 62021 | 672 | 33 | 9 | 4 | 5 | 8033 | 2018 | 1972 | 3 | 13 |
| Basset-Panama-CO1-S-ARM1-500-Dec2017_S5 | ARM1-DEC2017-500 | 361020 | 170164 | 222.8 | 36.8 | 44.3 | 61876 | 61876 | 46611 | 123125 | 428 | 35 | 9 | 8 | 3 | 8022 | 687 | 346 | 279 | 5 |
| Basset-Panama-CO1-S-ARM2-150-Dec2017_S6 | ARM2-DEC2017-150 | 386111 | 244040 | 226.7 | 36.7 | 42.0 | 72252 | 72252 | 6990 | 236609 | 441 | 34 | 6 | 9 | 3 | 9612 | 187 | 29 | 81 | 10 |
| Basset-Panama-CO1-S-ARM2-250-Dec2017_S7 | ARM2-DEC2017-250 | 410161 | 302207 | 233.1 | 36.8 | 36.2 | 75092 | 75092 | 57356 | 241466 | 3385 | 45 | 5 | 14 | 3 | 8393 | 1059 | 293 | 304 | 13 |
| Basset-Panama-CO1-S-ARM2-400-Dec2017_S8 | ARM2-DEC2017-400 | 403250 | 276021 | 240.8 | 36.4 | 39.5 | 81843 | 81843 | 2E+05 | 86817 | 5056 | 45 | 5 | 9 | 4 | 4420 | 1993 | 14 | 449 | 1281 |
| Basset-Panama-CO1-S-ARM2-450-Dec2017_S9 | ARM2-DEC2017-450 | 411140 | 281971 | 231.3 | 36.9 | 38.5 | 81472 | 81472 | 2E+05 | 96939 | 1598 | 38 | 6 | 7 | 3 | 8802 | 2033 | 330 | 534 | 5 |
| Basset-Panama-CO1-S-ARM2-500-Dec2017_S10 | ARM2-DEC2017-500 | 276125 | 158928 | 224.8 | 36.7 | 40.6 | 45277 | 45277 | 50060 | 108686 | 182 | 37 | 7 | 7 | 3 | 4472 | 533 | 23 | 29 | 35 |
| Basset-Panama-CO1-S-ARM3-050-Dec2017_S11 | ARM3-DEC2017-050 | 357280 | 189556 | 226.3 | 36.6 | 45.4 | 59128 | 59128 | 579 | 188023 | 954 | 28 | 3 | 8 | 4 | 7739 | 135 | 9 | 9 | 18 |
| Basset-Panama-CO1-S-ARM3-100-Dec2017_S12 | ARM3-DEC2017-100 | 224093 | 170111 | 231.1 | 36.7 | 42.6 | 28013 | 28013 | 2E+05 | 3939 | 94 | 25 | 5 | 8 | 3 | 2149 | 1649 | 26 | 8 | 7 |
| Basset-Panama-CO1-S-ARM3-150-Dec2017_S13 | ARM3-DEC2017-150 | 436557 | 298273 | 241.6 | 36.7 | 43.6 | 55248 | 55248 | 2E+05 | 97364 | 1403 | 23 | 6 | 2 | 3 | 3409 | 1482 | 25 | 1 | 9 |
| Basset-Panama-CO1-S-ARM3-300-Dec2017_S14 | ARM3-DEC2017-300 | 425947 | 299766 | 236.4 | 36.8 | 42.0 | 79967 | 79967 | 2E+05 | 121480 | 5355 | 44 | 6 | 6 | 12 | 7590 | 2673 | 42 | 35 | 2471 |
| Basset-Panama-CO1-S-ARM3-400-Dec2017_S15 | ARM3-DEC2017-400 | 428103 | 283058 | 240.5 | 36.8 | 44.9 | 53987 | 53987 | 2E+05 | 44163 | 607 | 20 | 4 | 5 | 3 | 3325 | 1034 | 17 | 10 | 9 |
| Basset-Panama-CO1-S-ARM4-100-Dec2017_S16 | ARM4-DEC2017-100 | 382534 | 273505 | 239.2 | 36.7 | 40.5 | 60013 | 60013 | 3603 | 269649 | 253 | 19 | 4 | 4 | 3 | 4215 | 146 | 82 | 1 | 8 |
| Basset-Panama-CO1-S-ARM4-250-Dec2017_S17 | ARM4-DEC2017-250 | 308792 | 257123 | 231.6 | 36.8 | 32.3 | 80520 | 80520 | 14064 | 241918 | 1141 | 28 | 8 | 7 | 5 | 6139 | 845 | 125 | 151 | 11 |
| Basset-Panama-CO1-S-ARM4-400-Dec2017_S18 | ARM4-DEC2017-400 | 233765 | 125954 | 227.8 | 36.7 | 42.9 | 39938 | 39938 | 17100 | 108756 | 100 | 17 | 3 | 3 | 2 | 2986 | 300 | 3 | 85 | 9 |
| Basset-Panama-CO1-S-ARM4-450-Dec2017_S19 | ARM4-DEC2017-450 | 558169 | 318627 | 242.5 | 36.6 | 50.0 | 46035 | 46035 | 864 | 317664 | 99 | 22 | 5 | 4 | 3 | 2576 | 115 | 21 | 8 | 30 |
| Basset-Panama-CO1-S-BAL1-050-Dec2017_S21 | BAL1-DEC2017-050 | 265207 | 182498 | 230.8 | 36.9 | 35.6 | 47365 | 47365 | 62314 | 115068 | 5116 | 20 | 4 | 5 | 2 | 3606 | 1232 | 94 | 14 | 12 |
| Basset-Panama-CO1-S-BAL1-100-Dec2017_S22 | BAL1-DEC2017-100 | 656333 | 420427 | 244.8 | 36.6 | 43.7 | 80090 | 80090 | 2E+05 | 171291 | 2459 | 25 | 2 | 1 | 9 | 4622 | 674 | 3 | 4 | 309 |
| Basset-Panama-CO1-S-BAL1-250-Dec2017_S23 | BAL1-DEC2017-250 | 96810 | 4959 | 236.5 | 36.1 | 41.2 | 1868 | 1868 | 98 | 4860 | 1 | 16 | 2 | 3 | 4 | 408 | 90 | 21 | 1 | 7 |
| Basset-Panama-CO1-S-BAL1-350-Dec2017_S24 | BAL1-DEC2017-350 | 339843 | 230497 | 231.6 | 36.5 | 35.7 | 73050 | 73050 | 95496 | 133660 | 1341 | 23 | 6 | 3 | 2 | 9109 | 1571 | 1258 | 225 | 5 |
| Basset-Panama-CO1-S-BAL1-400-Dec2017_S25 | BAL1-DEC2017-450 | 364556 | 211137 | 241.6 | 36.6 | 44.3 | 41602 | 41602 | 2E+05 | 31411 | 1691 | 28 | 5 | 9 | 1 | 2473 | 1270 | 30 | 13 | 3 |
| Basset-Panama-CO1-S-DRA1-50-Dec2017_S26 | DRA1-DEC2017-050 | 372222 | 237594 | 237.9 | 36.6 | 36.4 | 63454 | 63454 | 90820 | 141746 | 5028 | 26 | 3 | 8 | 4 | 4189 | 1718 | 1505 | 12 | 26 |
| Basset-Panama-CO1-S-DRA1-200-Dec2017_S27 | DRA1-DEC2017-200 | 356352 | 238724 | 240.1 | 36.6 | 37.8 | 80819 | 80819 | 28035 | 202498 | 8191 | 25 | 4 | 8 | 4 | 6504 | 580 | 63 | 10 | 32 |
| Basset-Panama-CO1-S-DRA1-250-Dec2017_S28 | DRA1-DEC2017-250 | 44933 | 341 | 236.5 | 32.6 | 35.9 | 329 | 329 | 67 | 269 | 5 | 18 | 3 | 5 | 3 | 218 | 48 | 20 | 2 | 1 |
| Basset-Panama-CO1-S-DRA1-300-Dec2017_S29 | DRA1-DEC2017-300 | 295217 | 175510 | 227.1 | 36.7 | 41.3 | 62828 | 62828 | 63330 | 111423 | 757 | 29 | 5 | 8 | 5 | 8358 | 962 | 115 | 205 | 103 |
| Basset-Panama-CO1-S-DRA1-350-Dec2017_S30 | DRA1-DEC2017-350 | 318378 | 230963 | 232.1 | 36.6 | 31.8 | 58478 | 58478 | 133 | 229210 | 1620 | 15 | 2 | 4 | 2 | 7058 | 621 | 20 | 10 | 4 |
| Basset-Panama-CO1-S-WHE1-50-Dec2017_S31 | WHE1-DEC2017-050 | 356510 | 245635 | 237.9 | 36.6 | 33.9 | 62459 | 62459 | 2E+05 | 32142 | 1248 | 26 | 2 | 7 | 3 | 4164 | 2052 | 1417 | 543 | 8 |
| Basset-Panama-CO1-S-WHE1-200-Dec2017_S32 | WHE1-DEC2017-200 | 332614 | 228347 | 233.9 | 36.7 | 34.0 | 68668 | 68668 | 1E+05 | 104955 | 1942 | 29 | 3 | 10 | 2 | 6089 | 2042 | 886 | 503 | 7 |
| Basset-Panama-CO1-S-WHE1-250-Dec2017_S33 | WHE1-DEC2017-250 | 207347 | 121524 | 233.4 | 36.6 | 34.1 | 34606 | 34606 | 61158 | 58951 | 1415 | 16 | 1 | 6 | 2 | 2266 | 1008 | 18 | 40 | 25 |
| Basset-Panama-CO1-S-WHE1-450-Dec2017_S35 | WHE1-DEC2017-450 | 166311 | 92385 | 236.3 | 36.7 | 43.0 | 19849 | 19849 | 41 | 92110 | 234 | 12 | 2 | 3 | 2 | 1346 | 44 | 6 | 2 | 0 |
| Basset-Panama-CO1-S-WHE2-50-Dec2017_S36 | WHE2-DEC2017-050 | 409042 | 283650 | 238.6 | 36.6 | 35.8 | 107891 | 107891 | 773 | 275256 | 7621 | 15 | 3 | 6 | 2 | 8590 | 274 | 27 | 14 | 7 |
| Basset-Panama-CO1-S-WHE2-100-Dec2017_S37 | WHE2-DEC2017-100 | 383994 | 245468 | 228.5 | 36.8 | 36.8 | 77361 | 77361 | 1E+05 | 110454 | 1208 | 36 | 13 | 4 | 2 | 10501 | 2023 | 1917 | 3 | 5 |
| Basset-Panama-CO1-S-ARM4-500-Dec2017_S20 | ARM4-DEC2017-500 | 233924 | 91089 | 228.9 | 36.7 | 50.4 | 34332 | 34332 | 10142 | 80643 | 304 | 17 | 2 | 4 | 5 | 2732 | 191 | 3 | 4 | 18 |
| Basset-Panama-CO1-S-WHE1-350-Dec2017_S34 | WHE1-DEC2017-350 | 394008 | 209273 | 238.0 | 36.6 | 37.5 | 72603 | 72603 | 67762 | 137205 | 4306 | 32 | 2 | 8 | 3 | 6851 | 1218 | 15 | 629 | 9 |
| Basset-Panama-CO1-S-WHE2-150-Dec2017_S38 | WHE2-DEC2017-150 | 410067 | 318918 | 236.3 | 36.8 | 31.5 | 77957 | 77957 | 3E+05 | 60832 | 2804 | 38 | 4 | 5 | 4 | 9596 | 2356 | 29 | 66 | 31 |
| Basset-Panama-CO1-S-WHE2-200-Dec2017_S39 | WHE2-DEC2017-200 | 372490 | 247515 | 230.0 | 36.8 | 37.5 | 82907 | 82907 | 1E+05 | 107587 | 1830 | 26 | 3 | 5 | 3 | 10838 | 1524 | 19 | 23 | 6 |
| Basset-Panama-CO1-S-WHE2-350-Dec2017_S40 | WHE2-DEC2017-350 | 340110 | 190302 | 237.6 | 36.1 | 38.2 | 59732 | 59732 | 1E+05 | 48083 | 391 | 24 | 6 | 2 | 3 | 4902 | 1560 | 25 | 3 | 79 |
| Basset-Panama-CO1-S-ZET1-150-Dec2017_S41 | ZET1-DEC2017-150 | 414374 | 292856 | 234.7 | 36.6 | 35.7 | 78600 | 78600 | 2E+05 | 109339 | 2299 | 39 | 9 | 10 | 3 | 7846 | 2198 | 1444 | 382 | 6 |
| Basset-Panama-CO1-S-ZET1-200-Dec2017_S42 | ZET1-DEC2017-200 | 121727 | 32602 | 231.0 | 36.7 | 35.7 | 7176 | 7176 | 31483 | 1097 | 22 | 18 | 4 | 2 | 2 | 767 | 470 | 24 | 2 | 17 |
| Basset-Panama-CO1-S-ZET1-300-Dec2017_S43 | ZET1-DEC2017-300 | 392413 | 252160 | 231.3 | 36.7 | 39.7 | 69343 | 69343 | 87897 | 163436 | 827 | 33 | 4 | 5 | 3 | 8530 | 1768 | 71 | 12 | 10 |
| Basset-Panama-CO1-S-ZET1-400-Dec2017_S44 | ZET1-DEC2017-400 | 501776 | 363534 | 229.9 | 36.8 | 37.4 | 84798 | 84798 | 2E+05 | 203928 | 3830 | 28 | 4 | 4 | 4 | 8268 | 1438 | 50 | 5 | 15 |
| Basset-Panama-CO1-S-ZET1-450-Dec2017_S45 | ZET1-DEC2017-450 | 528685 | 416775 | 241.2 | 36.8 | 38.1 | 88000 | 88000 | 1E+05 | 282492 | 6427 | 26 | 5 | 7 | 2 | 5954 | 970 | 22 | 26 | 9 |
| Basset-Panama-CO1-S-ZET2-100-Dec2017_S46 | ZET2-DEC2017-100 | 350044 | 243743 | 242.7 | 36.7 | 41.6 | 48292 | 48292 | 2E+05 | 90403 | 367 | 24 | 5 | 1 | 4 | 1606 | 1023 | 14 | 2 | 763 |
| Basset-Panama-CO1-S-ZET2-250-Dec2017_S47 | ZET2-DEC2017-250 | 328464 | 263871 | 230.8 | 36.9 | 30.6 | 44387 | 44387 | 1634 | 261661 | 576 | 22 | 3 | 3 | 5 | 4614 | 3813 | 21 | 8 | 10 |
| Basset-Panama-CO1-S-ZET2-300-Dec2017_S48 | ZET2-DEC2017-300 | 186743 | 161226 | 230.4 | 36.9 | 30.8 | 32575 | 32575 | 1E+05 | 36107 | 135 | 24 | 5 | 1 | 3 | 1883 | 1070 | 843 | 182 | 1 |
| Basset-Panama-F230R-S-B-ARM2-250-MAR17_S49 | ARM2-MAR2017-250 | 52042 | 6700 | 231.5 | 35.7 | 37.7 | 2852 | 2852 | 6560 | 136 | 4 | 31 | 0 | 1 | 2 | 119 | 100 | 0 | 0 | 17 |
| Basset-Panama-F230R-S-B-ARM2-350-MAR17_S50 | ARM2-MAR2017-350 | 159586 | 46340 | 232.1 | 35.7 | 40.9 | 15667 | 15667 | 39442 | 6679 | 219 | 18 | 0 | 1 | 2 | 500 | 453 | 0 | 2 | 403 |
| Basset-Panama-F230R-S-B-ARM3-300-MAR17_S51 | ARM3-MAR2017-350 | 41140 | 263 | 226.3 | 35.1 | 39.3 | 190 | 190 | 24 | 239 | 0 | 13 | 0 | 0 | 2 | 42 | 12 | 0 | 0 | 2 |
| Basset-Panama-F230R-S-B-ARM3-350-MAR17_S52 | ARM3-MAR2017-350 | 138106 | 15854 | 232.0 | 34.1 | 36.1 | 8824 | 8824 | 12565 | 3132 | 157 | 12 | 0 | 3 | 2 | 1056 | 978 | 0 | 20 | 3 |
| Basset-Panama-F230R-S-B-ARM3-400-MAR17_S53 | ARM3-MAR2017-400 | 211175 | 63345 | 231.7 | 35.6 | 40.1 | 21549 | 21549 | 7330 | 55731 | 284 | 20 | 0 | 1 | 5 | 1499 | 156 | 0 | 0 | 107 |
| Basset-Panama-F230R-S-B-ARM3-500-MAR17_S54 | ARM3-MAR2017-500 | 330024 | 94627 | 231.6 | 35.4 | 38.8 | 36895 | 36895 | 21792 | 71463 | 1372 | 21 | 1 | 2 | 2 | 2362 | 653 | 79 | 513 | 5 |
| Basset-Panama-F230R-S-B-ARM4-150-MAR17_S55 | ARM4-MAR2017-150 | 64705 | 3409 | 232.2 | 35.6 | 32.3 | 1963 | 1963 | 2516 | 877 | 16 | 20 | 3 | 3 | 3 | 161 | 121 | 5 | 14 | 3 |
| Basset-Panama-F230R-S-B-ARM4-200-MAR17_S56 | ARM4-MAR2017-200 | 56356 | 398 | 230.3 | 35.7 | 31.5 | 247 | 247 | 335 | 63 | 0 | 6 | 0 | 1 | 0 | 41 | 14 | 1 | 0 | 1 |
| Basset-Panama-F230R-S-B-ARM4-400-MAR17_S57 | ARM4-MAR2017-400 | 95334 | 6683 | 232.0 | 35.4 | 42.3 | 2697 | 2697 | 6535 | 144 | 4 | 18 | 1 | 1 | 1 | 91 | 70 | 1 | 4 | 0 |
| Basset-Panama-F230R-S-B-ARM4-450-MAR17_S58 | ARM4-MAR2017-450 | 114623 | 19155 | 231.9 | 35.5 | 35.2 | 8566 | 8566 | 112 | 18763 | 280 | 15 | 1 | 0 | 2 | 545 | 64 | 1 | 0 | 3 |
| Basset-Panama-F230R-S-B-ARM4-500-MAR17_S59 | ARM4-MAR2017-500 | 65130 | 970 | 234.2 | 35.4 | 37.0 | 531 | 531 | 492 | 478 | 0 | 12 | 0 | 0 | 2 | 33 | 20 | 0 | 0 | 0 |
| Basset-Panama-F230R-S-B-BAL1-50-MAR17_S60 | BAL1-MAR2017-050 | 119249 | 32193 | 231.9 | 36.0 | 33.2 | 7580 | 7580 | 23 | 32150 | 20 | 14 | 0 | 0 | 2 | 566 | 128 | 0 | 0 | 1 |
| Basset-Panama-F230R-S-B-BAL1-100-MAR17_S61 | BAL1-MAR2017-100 | 41930 | 93 | 231.9 | 34.1 | 37.6 | 91 | 91 | 19 | 73 | 1 | 10 | 2 | 1 | 3 | 30 | 13 | 1 | 0 | 1 |
| Basset-Panama-F230R-S-B-DRA1-100-MAR17_S62 | DRA1-MAR2017-100 | 237292 | 80940 | 229.4 | 35.5 | 34.6 | 34067 | 34067 | 43066 | 37015 | 859 | 22 | 3 | 2 | 3 | 2366 | 1628 | 782 | 1 | 467 |
| Basset-Panama-F230R-S-B-DRA1-150-MAR17_S63 | DRA1-MAR2017-150 | 231972 | 56409 | 229.8 | 35.3 | 34.8 | 20935 | 20935 | 19 | 55910 | 480 | 6 | 1 | 1 | 3 | 1389 | 101 | 29 | 1 | 7 |
| Basset-Panama-F230R-S-B-WHE1-50-MAR17_S64 | WHE1-MAR2017-050 | 315272 | 146099 | 232.4 | 35.6 | 38.4 | 49395 | 49395 | 1E+05 | 19679 | 2543 | 16 | 4 | 1 | 6 | 2177 | 1733 | 135 | 0 | 1095 |
| Basset-Panama-F230R-S-B-WHE1-200-MAR17_S65 | WHE1-MAR2017-200 | 183304 | 55227 | 231.8 | 35.8 | 30.8 | 21302 | 21302 | 24909 | 29638 | 680 | 14 | 3 | 1 | 4 | 1025 | 389 | 2 | 1 | 2 |
| Basset-Panama-F230R-S-B-WHE2-500-MAR17_S66_L001_R2_001 | WHE2-MAR2017-500 | 157858 | 53806 | 232.3 | 35.4 | 39.5 | 21479 | 21479 | 47521 | 5890 | 395 | 11 | 2 | 1 | 3 | 887 | 612 | 105 | 3 | 359 |
| Basset-Panama-F230R-S-B-ZET1-250-MAR17_S67 | ZET1-MAR2017-250 | 79644 | 2063 | 237.3 | 34.9 | 44.2 | 1053 | 1053 | 28 | 2033 | 2 | 8 | 4 | 1 | 1 | 68 | 19 | 4 | 0 | 4 |
| Basset-Panama-F230R-S-B-ZET1-350-MAR17_S68 | ZET1-MAR2017-350 | 312202 | 179078 | 231.9 | 35.8 | 33.8 | 57125 | 57125 | 15 | 175813 | 3250 | 8 | 2 | 0 | 2 | 4240 | 1234 | 2 | 0 | 10 |
| Basset-Panama-F230R-S-B-ZET1-500-MAR17_S69 | ZET1-MAR2017-500 | 354710 | 131887 | 232.4 | 35.7 | 30.0 | 45902 | 45902 | 51039 | 79532 | 1316 | 11 | 3 | 1 | 5 | 3163 | 1750 | 1174 | 209 | 3 |
| Basset-Panama-F230R-S-B-ZET2-50-MAR17_S70 | ZET2-MAR2017-050 | 320036 | 128549 | 232.4 | 35.4 | 31.7 | 43618 | 43618 | 15435 | 111802 | 1312 | 10 | 2 | 2 | 3 | 3045 | 1902 | 260 | 4 | 13 |
| Basset-Panama-F230R-S-B-ZET2-300-MAR17_S71 | ZET2-MAR2017-300 | 302320 | 106102 | 232.4 | 35.4 | 30.2 | 34071 | 34071 | 1E+05 | 3534 | 297 | 11 | 4 | 2 | 3 | 1778 | 1581 | 1233 | 319 | 4 |
| Basset-Panama-F230R-S-B-ZET2-450-MAR17_S72 | ZET2-MAR2017-450 | 203286 | 51787 | 232.4 | 35.4 | 40.8 | 18858 | 18858 | 25695 | 25778 | 314 | 8 | 1 | 0 | 6 | 1173 | 451 | 3 | 0 | 430 |
| Basset-Panama-F230R-S-B-ARM2-200-MAR17_S48 | ARM2-MAR2017-200 | 88428 | 9369 | 228.5 | 35.2 | 38.8 | 3598 | 3598 | 37 | 9328 | 4 | 21 | 1 | 0 | 0 | 191 | 24 | 0 | 0 | 0 |
| Basset-Panama-F230R-S-B-ARM2-50-MAR17_S47 | ARM2-MAR2017-050 | 48716 | 79 | 220.4 | 33.4 | 35.1 | 79 | 79 | 33 | 46 | 0 | 21 | 1 | 0 | 0 | 44 | 29 | 2 | 0 | 0 |
| Basset-Panama-F230R-S-B-ARM1-500-MAR17_S46 | ARM1-MAR2017-500 | 187242 | 38349 | 230.7 | 35.6 | 35.0 | 19160 | 19160 | 17041 | 20115 | 1193 | 22 | 3 | 5 | 1 | 1112 | 434 | 154 | 134 | 4 |
| Basset-Panama-F230R-S-B-ARM1-450-MAR17_S45 | ARM1-MAR2017-450 | 74782 | 8945 | 232.3 | 35.4 | 29.0 | 3970 | 3970 | 8466 | 464 | 15 | 28 | 2 | 0 | 0 | 402 | 369 | 348 | 0 | 0 |
| Basset-Panama-F230R-S-B-ARM1-350-MAR17_S44 | ARM1-MAR2017-350 | 102481 | 11776 | 230.7 | 35.7 | 29.0 | 4724 | 4724 | 242 | 11497 | 37 | 14 | 0 | 0 | 0 | 295 | 35 | 0 | 0 | 0 |
| Basset-Panama-F230R-S-B-ARM1-50-MAR17_S43 | ARM1-MAR2017-050 | 139126 | 48688 | 232.2 | 35.7 | 36.7 | 15101 | 15101 | 47 | 48571 | 70 | 26 | 2 | 0 | 0 | 1307 | 228 | 0 | 0 | 3 |
| Basset-Panama-CO1-S-ZET2-500-Dec2017_S50 | ZET2-DEC2017-500 | 395552 | 298422 | 238.9 | 36.7 | 39.9 | 64047 | 64047 | 3E+05 | 37558 | 548 | 12 | 3 | 2 | 2 | 3243 | 2237 | 111 | 47 | 2039 |
| Basset-Panama-CO1-S-ZET2-350-Dec2017_S49 | ZET2-DEC2017-350 | 426174 | 267654 | 236.7 | 36.7 | 44.7 | 59149 | 59149 | 639 | 265158 | 1857 | 12 | 2 | 1 | 1 | 4146 | 101 | 6 | 6 | 16 |

^1^Including singletons. See Appendix S2 and Table S6 for more details.

**Table S5.** Number of reads in BINs (first entry in location columns), number of unique BINs (second entry) and number of reads in OTUs (third entry) for arthropod orders detected by metabarcoding, detailed by sampling location.

| **CLASS/Order** | **Reads in BINs** | **BINs** | **Reads in OTUs** | **ARM1** | **ARM2** | **ARM3** | **ARM4** | **BAL1** | **DRA1** | **WHE1** | **WHE2** | **ZET1** | **ZET2** |
| --- | --- | --- | --- | --- | --- | --- | --- | --- | --- | --- | --- | --- | --- |
| **ARACHNIDA** |  |  |  |  |  |  |  |  |  |  |  |  |  |
| Araneae | 25013 | 9 | 316097 | 71/1/2763 | 748/3/94 | 8/3/100 | 327/2/30847 | 3/2/240 | 11/4/1263 | 23814/3/70691 | 12/5/553 | 12/5/209506 | 7/2/40 |
| Ixodida | 0 | 0 | 8 | 0/0/2 | 0/0/2 | 0/0/0 | 0/0/0 | 0/0/0 | 0/0/2 | 0/0/0 | 0/0/1 | 0/0/0 | 0/0/1 |
| Mesostigmata | 782 | 4 | 11364 | 0/0/0 | 502/1/323 | 0/0/3412 | 0/0/4777 | 0/0/0 | 0/0/2833 | 0/0/0 | 99/2/11 | 181/2/8 | 0/0/0 |
| Opiliones | 7594 | 1 | 63395 | 2/1/1 | 0/0/5 | 3034/1/92 | 4521/3/1522 | 0/0/61773 | 0/0/1 | 37/1/0 | 0/0/1 | 0/0/0 | 0/0/0 |
| Sarcoptiformes | 2964 | 4 | 4767 | 0/0/0 | 0/0/36 | 38/1/1 | 807/1/2222 | 27/1/0 | 0/0/0 | 90/1/1206 | 1295/1/107 | 706/4/1195 | 1/1/0 |
| Schizomida | 0 | 0 | 49390 | 0/0/0 | 0/0/32660 | 0/0/2 | 0/0/1 | 0/0/34 | 0/0/220 | 0/0/0 | 0/0/0 | 0/0/0 | 0/0/16473 |
| Scorpiones | 0 | 0 | 15 | 0/0/0 | 0/0/0 | 0/0/1 | 0/0/2 | 0/0/0 | 0/0/0 | 0/0/0 | 0/0/9 | 0/0/3 | 0/0/0 |
| Solifugae | 0 | 0 | 1 | 0/0/0 | 0/0/0 | 0/0/0 | 0/0/0 | 0/0/0 | 0/0/1 | 0/0/0 | 0/0/0 | 0/0/0 | 0/0/0 |
| Trombidiformes | 456 | 3 | 4346 | 2/2/405 | 2/1/1 | 452/3/1666 | 0/0/0 | 0/0/0 | 0/0/0 | 0/0/183 | 0/0/1931 | 0/0/157 | 0/0/3 |
| **BRANCHIOPODA** |  |  |  |  |  |  |  |  |  |  |  |  |  |
| Anomopoda | 1 | 1 | 773 | 0/0/3 | 0/0/2 | 0/0/1 | 0/0/2 | 1/1/626 | 0/0/3 | 0/0/24 | 0/0/8 | 0/0/98 | 0/0/6 |
| Ctenopoda | 2 | 1 | 0 | 0/0/0 | 0/0/0 | 0/0/0 | 0/0/0 | 0/0/0 | 0/0/0 | 0/0/0 | 0/0/0 | 2/1/0 | 0/0/0 |
| Notostraca | 0 | 0 | 6 | 0/0/0 | 0/0/0 | 0/0/0 | 0/0/0 | 0/0/0 | 0/0/1 | 0/0/1 | 0/0/0 | 0/0/3 | 0/0/1 |
| **CHILOPODA** |  |  |  |  |  |  |  |  |  |  |  |  |  |
| Geophilomorpha | 0 | 0 | 1 | 0/0/1 | 0/0/0 | 0/0/0 | 0/0/0 | 0/0/0 | 0/0/0 | 0/0/0 | 0/0/0 | 0/0/0 | 0/0/0 |
| Lithobiomorpha | 0 | 0 | 8686 | 0/0/0 | 0/0/0 | 0/0/0 | 0/0/0 | 0/0/2 | 0/0/0 | 0/0/0 | 0/0/8684 | 0/0/0 | 0/0/0 |
| Polydesmida | 0 | 0 | 745 | 0/0/0 | 0/0/0 | 0/0/0 | 0/0/0 | 0/0/0 | 0/0/0 | 0/0/0 | 0/0/0 | 0/0/745 | 0/0/0 |
| Scolopendromorpha | 1 | 1 | 6476 | 0/0/6 | 0/0/0 | 0/0/51 | 0/0/4 | 0/0/4871 | 1/1/2 | 0/0/203 | 0/0/693 | 0/0/644 | 0/0/2 |
| Scutigeromorpha | 0 | 0 | 2 | 0/0/1 | 0/0/1 | 0/0/0 | 0/0/0 | 0/0/0 | 0/0/0 | 0/0/0 | 0/0/0 | 0/0/0 | 0/0/0 |
| **COLLEMBOLA** |  |  |  |  |  |  |  |  |  |  |  |  |  |
| Entomobryomorpha | 252019 | 25 | 91379 | 22222/23/14798 | 47753/26/13574 | 3049/26/277 | 12812/19/7614 | 426/12/10116 | 12457/22/415 | 82409/30/25438 | 4180/11/423 | 34683/20/17320 | 32028/8/1404 |
| Neelipleona | 553 | 1 | 16 | 0/0/0 | 0/0/0 | 13/1/0 | 0/0/0 | 0/0/0 | 484/3/14 | 0/0/0 | 0/0/0 | 56/1/2 | 0/0/0 |
| Poduromorpha | 86223 | 6 | 14418 | 812/6/1173 | 1666/6/1294 | 16562/4/1471 | 2502/9/4336 | 12383/5/1322 | 10/6/422 | 37944/9/2289 | 3/3/9 | 6798/6/1651 | 7543/8/451 |
| Symphypleona | 54023 | 5 | 8455 | 934/11/56 | 36371/30/6406 | 625/10/96 | 6/4/6 | 5551/8/357 | 448/8/29 | 7937/10/852 | 2087/12/633 | 64/7/16 | 0/0/4 |
| **INSECTA** |  |  |  |  |  |  |  |  |  |  |  |  |  |
| Archaeognatha | 0 | 0 | 751 | 0/0/376 | 0/0/0 | 0/0/0 | 0/0/0 | 0/0/0 | 0/0/0 | 0/0/0 | 0/0/1 | 0/0/374 | 0/0/0 |
| Coleoptera | 1238676 | 47 | 1248645 | 210062/47/98854 | 244059/56/20726 | 52852/46/162966 | 4683/20/162720 | 5771/19/95263 | 44611/22/104830 | 16409/20/51085 | 403608/34/31080 | 239476/38/411815 | 17145/27/109306 |
| Dicellurata | 0 | 0 | 1285 | 0/0/0 | 0/0/0 | 0/0/0 | 0/0/0 | 0/0/0 | 0/0/0 | 0/0/0 | 0/0/1282 | 0/0/1 | 0/0/2 |
| Diptera | 256057 | 128 | 348213 | 39086/62/579 | 25955/80/1798 | 1115/30/300 | 23845/52/11628 | 51837/23/4272 | 572/16/5194 | 72288/16/2686 | 7732/21/54370 | 31814/18/5068 | 1813/12/262318 |
| Ephemeroptera | 114 | 12 | 42 | 23/14/14 | 26/9/14 | 14/6/0 | 2/1/3 | 2/2/2 | 4/3/2 | 3/2/0 | 0/0/0 | 0/0/0 | 40/2/7 |
| Hemiptera | 58286 | 20 | 296791 | 869/8/3985 | 8412/13/2305 | 4/2/1299 | 1/1/16163 | 8/6/42145 | 42/5/218140 | 35471/12/1531 | 13324/6/942 | 8/4/1074 | 147/5/9207 |
| Hymenoptera | 1411049 | 53 | 270366 | 317662/51/17688 | 44128/34/58841 | 12499/28/1910 | 6278/33/824 | 123968/25/3503 | 114277/24/80600 | 226037/20/42067 | 138740/40/8978 | 168605/42/41297 | 258855/32/14658 |
| Isoptera | 1669489 | 35 | 196929 | 115314/31/7176 | 236396/37/40370 | 242653/55/75218 | 1393/36/96 | 202455/32/3635 | 170860/31/19322 | 106767/30/15082 | 184800/27/4490 | 921/31/85 | 407930/33/31455 |
| Lepidoptera | 782 | 4 | 87543 | 0/0/245 | 11/2/78 | 661/2/21 | 99/2/32 | 1/1/335 | 0/0/31575 | 0/0/21652 | 0/0/29900 | 0/0/3636 | 10/2/69 |
| Mantodea | 0 | 0 | 15003 | 0/0/2 | 0/0/1 | 0/0/0 | 0/0/0 | 0/0/21 | 0/0/14471 | 0/0/2 | 0/0/0 | 0/0/343 | 0/0/163 |
| Mecoptera | 0 | 0 | 2 | 0/0/0 | 0/0/0 | 0/0/0 | 0/0/0 | 0/0/0 | 0/0/0 | 0/0/0 | 0/0/1 | 0/0/1 | 0/0/0 |
| Megaloptera | 0 | 0 | 11 | 0/0/0 | 0/0/0 | 0/0/0 | 0/0/0 | 0/0/0 | 0/0/0 | 0/0/0 | 0/0/0 | 0/0/11 | 0/0/0 |
| Odonata | 7 | 3 | 10450 | 0/0/10 | 4/1/1 | 0/0/0 | 1/1/0 | 2/1/0 | 0/0/1 | 0/0/2 | 0/0/10289 | 0/0/146 | 0/0/1 |
| Orthoptera | 398 | 1 | 42783 | 0/0/0 | 0/0/18 | 0/0/9 | 0/0/29 | 0/0/31702 | 0/0/21 | 398/1/40 | 0/0/8 | 0/0/10933 | 0/0/23 |
| Psocodea | 118347 | 5 | 2468 | 0/0/1 | 0/0/2 | 464/4/49 | 18/5/3 | 38706/4/1177 | 1/1/2 | 3/2/3 | 51839/8/892 | 27305/11/334 | 11/5/5 |
| Thysanoptera | 4 | 2 | 0 | 2/2/0 | 2/2/0 | 0/0/0 | 0/0/0 | 0/0/0 | 0/0/0 | 0/0/0 | 0/0/0 | 0/0/0 | 0/0/0 |
| Trichoptera | 14 | 8 | 2 | 2/2/0 | 1/1/0 | 0/0/0 | 7/5/1 | 0/0/0 | 0/0/0 | 0/0/0 | 2/1/0 | 0/0/0 | 2/1/1 |
|  |  |  |  |  |  |  |  |  |  |  |  |  |  |
| **OSTRACODA** |  |  |  |  |  |  |  |  |  |  |  |  |  |
| Podocopida | 0 | 0 | 7 | 0/0/0 | 0/0/0 | 0/0/0 | 0/0/2 | 0/0/0 | 0/0/0 | 0/0/0 | 0/0/5 | 0/0/0 | 0/0/0 |
| **MALACOSTRACA** |  |  |  |  |  |  |  |  |  |  |  |  |  |
| Amphipoda | 15 | 5 | 47314 | 1/1/92 | 3/2/1090 | 2/1/20120 | 0/0/567 | 3/2/47 | 3/3/0 | 0/0/0 | 1/1/22063 | 2/2/3335 | 0/0/0 |
| Cumacea | 0 | 0 | 2 | 0/0/0 | 0/0/0 | 0/0/0 | 0/0/0 | 0/0/2 | 0/0/0 | 0/0/0 | 0/0/0 | 0/0/0 | 0/0/0 |
| Decapoda | 22 | 4 | 147822 | 8/3/1683 | 6/2/232 | 8/4/66590 | 0/0/6089 | 0/0/36749 | 0/0/64 | 0/0/5639 | 0/0/3155 | 0/0/5341 | 0/0/22280 |
| Isopoda | 981643 | 2 | 106047 | 154/5/41 | 156/6/37 | 594962/8/6078 | 78512/9/262 | 181171/8/28353 | 1728/7/67832 | 76/6/15 | 78/5/31 | 124773/5/3380 | 33/5/18 |
| Mysida | 0 | 0 | 54 | 0/0/0 | 0/0/0 | 0/0/0 | 0/0/0 | 0/0/0 | 0/0/0 | 0/0/54 | 0/0/0 | 0/0/0 | 0/0/0 |

**Table S6.** Number of OTUs and complexes of focal species detected for three treatments: no filter, filter with a minimum of 0.003% of total OTU sequences, and minimum of 0.005% of total OTU sequences. See Appendix S2 for discussion.

| **Variable** | **Taxa** | **No filter** | **Filter > 228 sequences** | **Filter > 379 sequences** |
| --- | --- | --- | --- | --- |
|  |  |  | **(0.003% of sequences)** | **(0.005% of sequences)** |
| OTU (n) | Arthropoda | 78853 | 576 | 429 |
| OTU (n) | Formicidae | 18361 | 63 | 47 |
| Complex of species (n)^1^ | Formicidae | 60 | 8 | 5 |
| Main species targeted (% total OTUs) | Formicidae | *Tranopelta gilva* (56) | *Tranopelta gilva* (87) | *Tranopelta gilva* (91) |
| OTU (n) | Collembola | 6255 | 77 | 52 |
| Complex of species (n)^1^ | Collembola | 32 | 28 | 19 |
| Main species targeted (% total OTUs) | Collembola | *Isotomurus retardatus* (16),  *Dicranocentrus marias* (16) | *Isotomurus retardatus* (45),  *Dicranocentrus marias* (12) | *Isotomurus retardatus* (46),  *Dicranocentrus marias* (15) |
| OTU (n) | Isoptera | 11679 | 99 | 78 |
| Complex of species (n)^1^ | Isoptera | 149 | 38 | 35 |
| Main species targeted (% total OTUs) | Isoptera | Apicotermitinae A^2^ (23),  Apicotermitinae B (11) | Apicotermitinae A (17),  Apicotermitinae B (10) | Apicotermitinae A (17),  Apicotermitinae B (8) |

^1^Similarity 90-97% to target species

^2^The two target termite species are yet undescribed

**Table S7.** In silico PCR results using EcoPCR. Forward primer: CCIGAYATRGCITTYCCICG. Reverse primer: CCIGTIYTIGCIGGIGCIATYAC.

| Probable false negative species (Genbank COI barcodes): | *In silico* amplification | Missmatch (F/R) | Sequence length |
| --- | --- | --- | --- |
| *Americanura basseti* | yes | 3/6 | 310 |
| *Folsomia sensibilis* | yes | 3/6 | 310 |
| *Isotomodes falsus* | yes | 3/6 | 310 |
| *Mesaphorura yosii* | yes | 3/6 | 310 |
| *Octostruma amrishi* | yes | 4/6 | 310 |
| *Pseudachorutes* sp. AAG1117 | yes | 3/6 | 310 |
| *Solenopsis* sp. AAN9169 | yes | 12/15 | 310 |
| *Solenopsis* sp. AAP9747 | yes | 13/14 | 310 |
| *Solenopsis* sp. AAZ7574 | yes | 4/6 | 310 |
| *Willemia panamaensis* | yes | 3/6 | 310 |


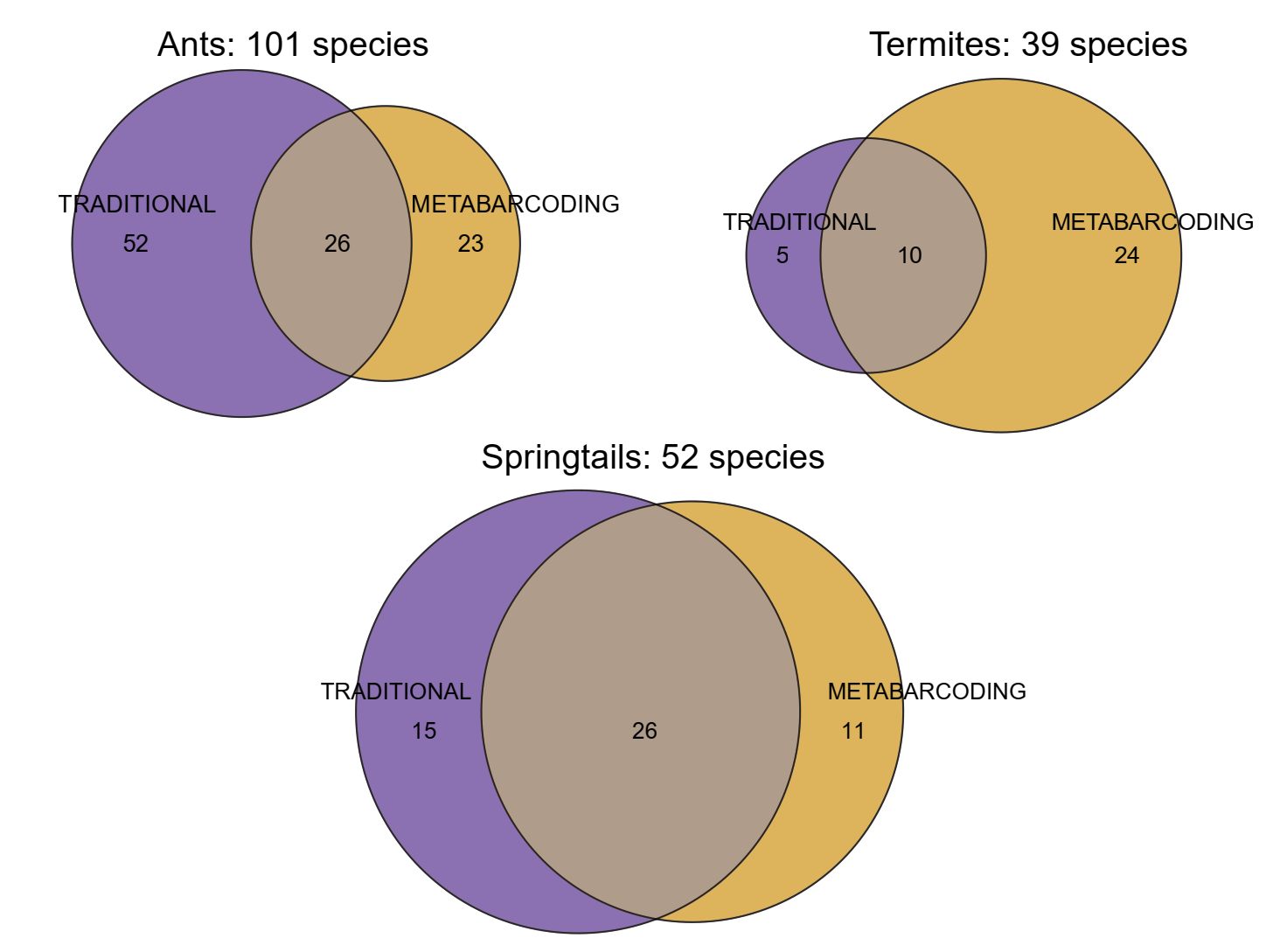
**Fig. S1.** Euler diagrams indicating the number of species with BINs detected by traditional and metabarcoding samples, and by both methods for ants, springtails and termites.


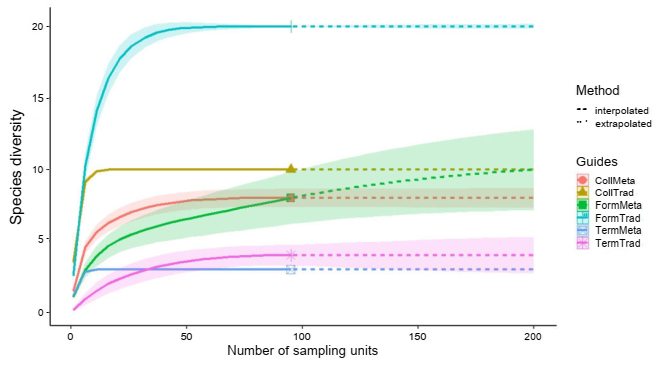


**Fig. S2.** Accumulation of species richness vs. the number of samples for common species of ants, springtails and termites, detailed for traditional and metabarcoding samples. Presentation as in Fig. 2.


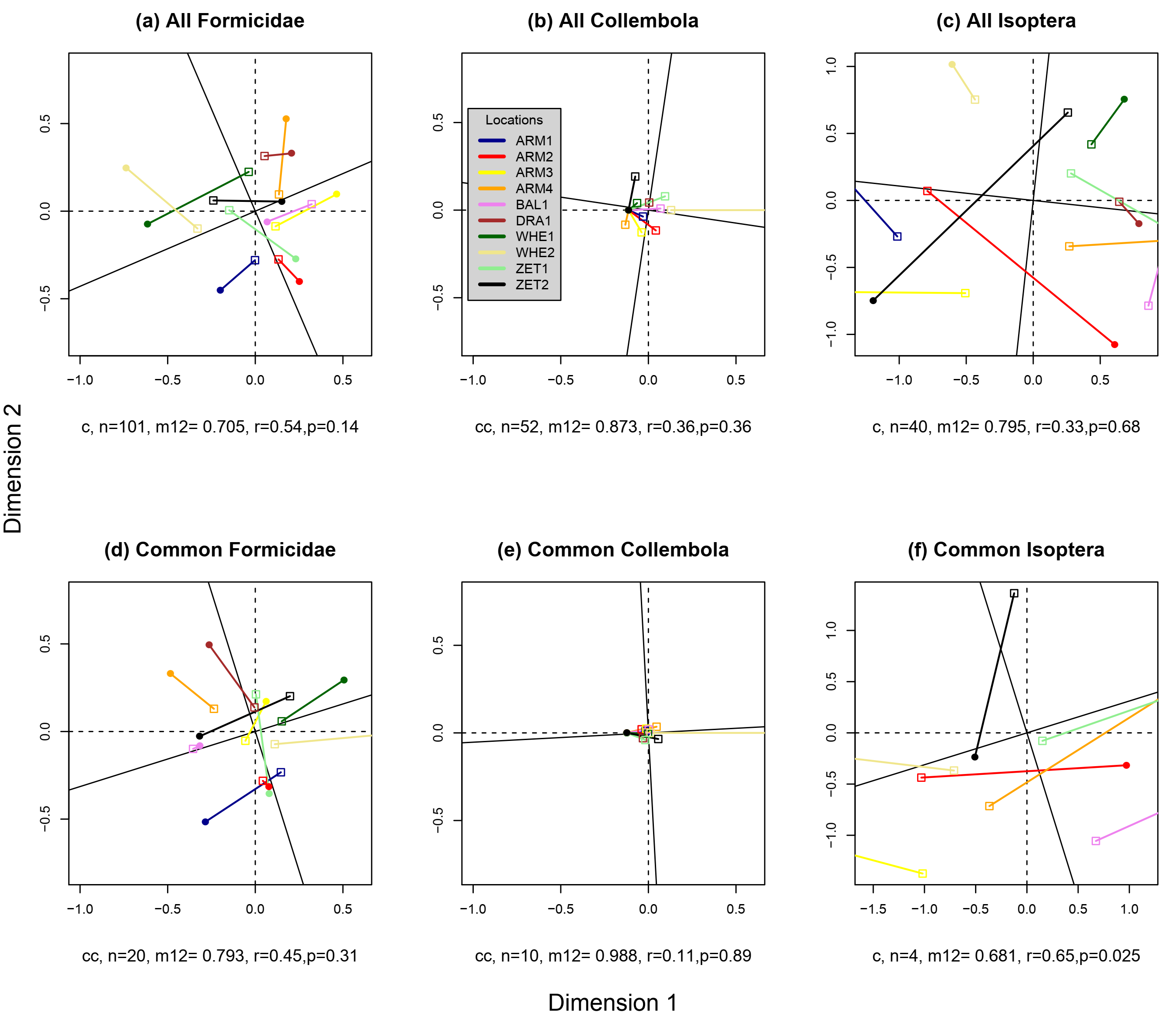


**Fig. S3.** Plot of sample locations (legend in panel b) in the first two axes of the Procrustes rotation for all species of Formicidae, Collembola and Isoptera (a-c) and for common species of the same taxa (d-f). The position of the locations in the ordination for traditional data are indicated by open squares, the position of locations in the target ordination, the metabarcoding data, by solid circles. Entries are whether the rotation is clockwise (c) or counter-clockwise (cc); n: total number of species considered for the analysis; m12: m12 squared from protest; r: correlation coefficient; p: probability. The plot also shows the rotation between the two ordinations (solid black lines) necessary to make them match as closely as possible.
